# Mechanism of Lian-Huo-Hua-Zhuo Formula in Alleviating Gastric Mucosal Inflammation in a Mouse Model of Chronic Atrophic Gastritis by Inhibiting the IL-17 Signaling Pathway

**DOI:** 10.3390/ph19071043

**Published:** 2026-07-05

**Authors:** Xiaoxuan Mo, Fan Gao, Jiaye Tian, Fengyue Xu, Zeyang Xie, Hongyan Wei, Jinhu Yang, Jianming Jiang, Guoxing Deng, Qiuhong Guo

**Affiliations:** 1 School of Traditional Chinese Medicine, Hebei University of Chinese Medicine, Shijiazhuang 050200, China; yjs20232015@hebcm.edu.cn (X.M.); yjs20231004@hebcm.edu.cn (F.G.); yjs20241004@hebcm.edu.cn (J.T.); yjs20232114@hebcm.edu.cn (F.X.); yjs20232123@hebcm.edu.cn (Z.X.); yjs20242012@hebcm.edu.cn (H.W.); yjs20242029@hebcm.edu.cn (J.Y.); jjmtgzy@126.com (J.J.); 2 Hebei Technology Innovation Center of TCM Formula Preparations, Shijiazhuang 050200, China

**Keywords:** chronic atrophic gastritis, gut microbiota, IL-17 pathway, Lian-Huo-Hua-Zhuo formula, short-chain fatty acids

## Abstract

**Background**: Chronic atrophic gastritis (CAG) is a prevalent precancerous gastric disorder characterized by persistent inflammation, glandular atrophy, and progressive mucosal damage, for which effective multi-target therapeutic strategies remain insufficient. The Lian-Huo-Hua-Zhuo formula (LHHZ), a traditional Chinese herbal prescription, has demonstrated potential anti-inflammatory and gastrointestinal protective effects in clinical practice; however, its active constituents and mechanisms of action against CAG remain undefined. This study aimed to clarify the absorbed bioactive components of LHHZ and explore its therapeutic mechanism for CAG. **Methods**: Ultra-high-performance liquid chromatography coupled with quadrupole Orbitrap high-resolution mass spectrometry was employed to identify the absorbed components of LHHZ in the gastric and intestinal tissues of mice. The therapeutic effects of LHHZ on CAG were assessed through histopathological staining, ultrastructural observation, and evaluation of serum and gastric functional indicators. Network pharmacology, molecular docking, and molecular dynamics simulations were integrated to predict the core targets and key signaling pathways, while the regulatory effects on the interleukin-17 (IL-17) signaling pathway were further validated by immunofluorescence staining, real-time quantitative polymerase chain reaction, and Western blotting. Additionally, 16S ribosomal RNA gene sequencing and targeted metabolomics were applied to investigate the effects of LHHZ on gut microbiota composition and short-chain fatty acid (SCFA) metabolism. **Results**: The results revealed that 55 and 48 absorbed components were identified in the gastric and intestinal tissues, respectively, predominantly derived from *Coptis chinensis* Franch. and *Pogostemon cablin* (Blanco) Benth. LHHZ significantly alleviated gastric mucosal lesions, reduced intestinal metaplasia, restored the ultrastructure of gastric mucosal cells, improved gastric functional indicators including pepsinogen I (PG I), pepsinogen II (PG II), and gastrin-17 (GAS-17), and decreased the levels of pro-inflammatory cytokines. Network pharmacology combined with in vitro and in vivo experiments demonstrated that the core bioactive components of LHHZ can target and regulate interleukin-1 beta (IL-1β) and tumor necrosis factor-alpha (TNF-α), attenuate activation of the IL-17 signaling pathway, and suppress the secretion of downstream pro-inflammatory factors. Furthermore, LHHZ enhanced the alpha diversity of gut microbiota, reduced the Firmicutes to Bacteroidetes (F/B) ratio, restored the abundance of SCFA-producing bacteria such as Bacteroidales and Oscillospirales, and normalized the aberrant levels of eight SCFAs. Significant correlations were also observed between gut microbiota composition and SCFA metabolism. **Conclusions**: These findings suggest that LHHZ alleviates CAG by inhibiting inflammation via the IL-17 signaling pathway and by modulating the gut microbiota–SCFA axis, thereby providing preclinical evidence supporting its further investigation and development for multi-target therapeutic strategies against CAG.

## 1. Introduction

Chronic atrophic gastritis (CAG) is a prevalent precancerous gastric disorder characterized by gastric mucosal atrophy, glandular hypoplasia, and intestinal metaplasia [1]. It is recognized as a critical stage in the progression from chronic inflammation to gastric cancer, consistent with the Correa model of gastric carcinogenesis [2,3]. Epidemiological evidence indicates that patients with CAG exhibit an elevated risk of developing gastric cancer, particularly among Asian populations, including those in China [4]. Consequently, early intervention in CAG is of substantial importance for preventing malignant transformation.

Current clinical management of CAG primarily involves acid suppressants, prokinetic agents, *Helicobacter pylori* eradication therapy, and gastrin modulators [5]. Nonetheless, these interventions often confer limited long-term benefit and are insufficient to fully reverse pathological alterations such as gastric mucosal atrophy and intestinal metaplasia. Therefore, the development of safer and more effective therapeutic strategies for CAG remains necessary.

Clinical evidence of traditional Chinese medicine for CAG.

Traditional Chinese medicine (TCM) has been widely used in the management of CAG. Accumulating clinical studies suggest that TCM may provide potential benefits in symptom relief, endoscopic findings, and serological markers in patients with CAG [6,7,8,9,10]. Some randomized and controlled clinical studies have reported that certain TCM formulas, used alone or in combination with conventional therapies, may improve clinical symptoms, endoscopic findings, and histopathological changes in patients with CAG [6,7]. Meta-analyses have further suggested that TCM interventions may be associated with higher total effective rates, improved regulation of the serum pepsinogen I/II ratio and gastrin-17 levels, and lower recurrence rates [8,9]. For example, a recent systematic review reported that TCM combined with conventional treatment was associated with a significantly higher overall response rate than conventional treatment alone [10]. However, the included studies varied in methodological quality, and the statistical robustness of the evidence remains limited. Taken together, these findings suggest that TCM may have potential value in symptom relief and disease management in CAG; however, the current evidence remains insufficient to draw definitive conclusions regarding its ability to delay or reverse pathological progression. Meanwhile, the pharmacodynamic material basis and molecular mechanisms of many TCM formulas remain largely undefined.

The Lian-Huo-Hua-Zhuo Formula (LHHZ) is a simplified derivative of the clinically utilized Xiang-Lian-Hua-Zhuo Formula (XLHZ), which has been employed in the management of chronic gastritis and CAG in Hebei Province [11,12]. Existing reports and clinical practice experience suggest that LHHZ/XLHZ may have potential benefits in ameliorating gastrointestinal symptoms, modulating gastrointestinal hormones, and promoting mucosal recovery in patients with chronic gastritis and CAG [13,14]. However, high-quality randomized controlled trials specifically evaluating LHHZ are currently lacking. Therefore, its clinical efficacy has not yet been conclusively established, and its pharmacodynamic material basis and underlying mechanisms remain unclear.

Chronic inflammation is a critical driving factor in the progression of CAG [15]. Among the relevant inflammatory pathways, the interleukin-17 (IL-17) signaling pathway has been reported to promote the secretion of pro-inflammatory mediators, compromise gastric mucosal barrier integrity, and exacerbate glandular atrophy [16]. Furthermore, accumulating evidence suggests that gut microbiota (GM) dysbiosis and altered short-chain fatty acid (SCFA) metabolism are closely associated with mucosal inflammation and disease progression in gastrointestinal disorders [17]. Nevertheless, whether LHHZ exerts anti-CAG effects via modulation of the IL-17 signaling pathway and the GM–SCFA axis remains unclear.

In the present study, we identified the absorbed components of LHHZ in gastrointestinal tissues and evaluated its therapeutic effects in CAG mice. We further integrated network pharmacology, molecular docking, molecular dynamics simulations, and experimental validation to investigate its regulatory effects on the IL-17 signaling pathway. Additionally, we explored the effects of LHHZ on GM composition and SCFA metabolism. This study aimed to elucidate the material basis and potential mechanisms of LHHZ against CAG and to provide experimental evidence for its further preclinical evaluation.

## 2. Results

### 2.1. Pharmacodynamic Material Basis of LHHZ and Analysis of Components In Vivo Gastrointestinal Tissues

**Figure 1:**
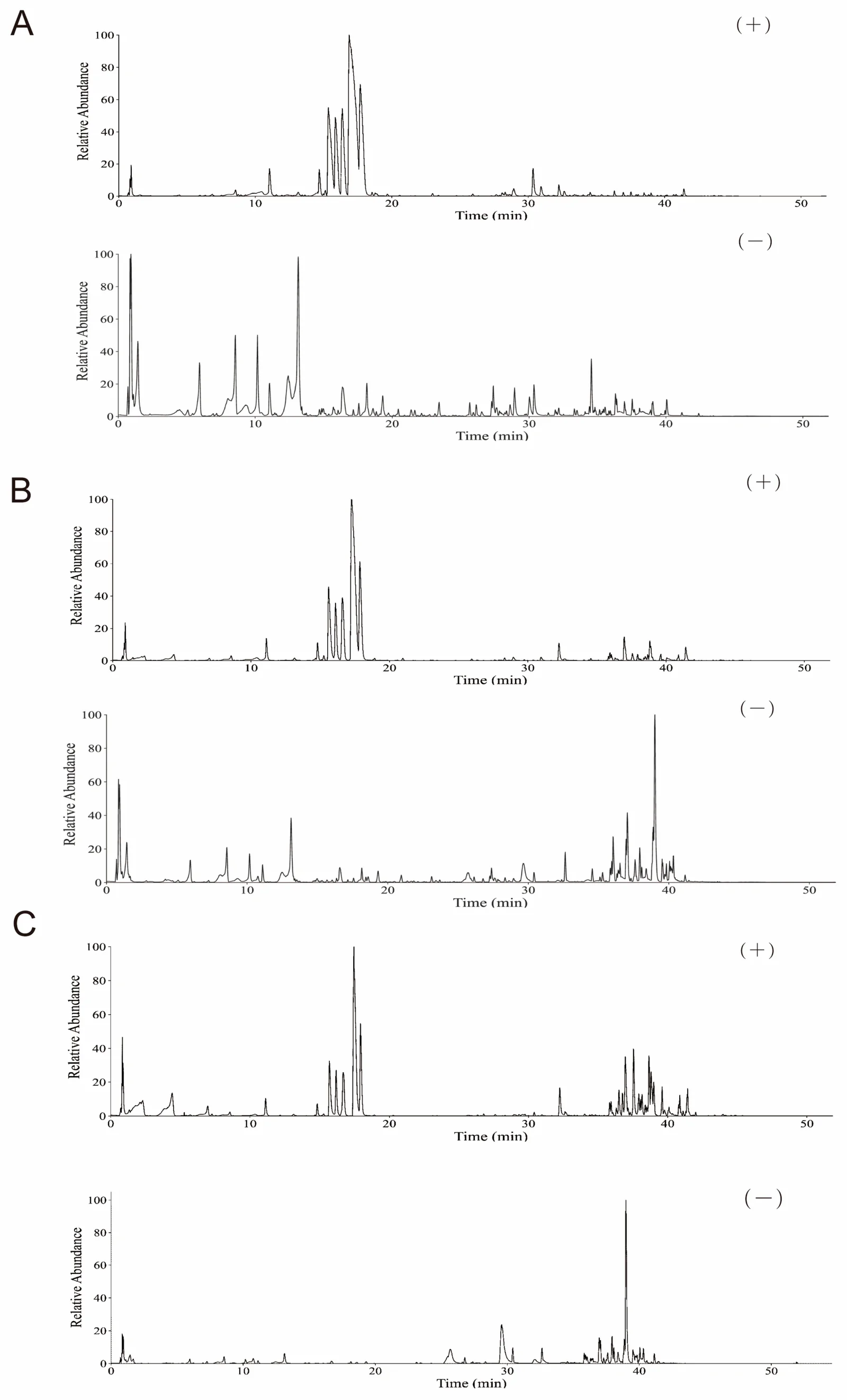
Total ion current (TIC) chromatograms acquired by ultra-high performance liquid chromatography coupled with Q-Exactive orbitrap high-resolution mass spectrometry (UHPLC-Q-Orbitrap HRMS) in positive and negative electrospray ionization modes. (**A**) Chemical profiles of the Lianhua Huazhuo (LHHZ) traditional Chinese medicine compound; (**B**) chemical components detected in gastric tissue samples; (**C**) chemical components detected in intestinal tissue samples.

Clarifying the chemical composition of LHHZ is a prerequisite and fundamental basis for screening its pharmacodynamic material basis and evaluating its quality. Therefore, we first analyzed and identified the chemical components of LHHZ using ultra-high-performance liquid chromatography coupled with quadrupole Orbitrap high-resolution mass spectrometry (UHPLC-Q-Orbitrap HRMS) (Figure 1A). The analysis identified a total of 85 chemical constituents in LHHZ (Supplementary Table S1-1), including 27 components derived from *Coptis chinensis* Franch., 34 from *Pogostemon cablin* (Blanco) Benth., and 27 from *Lilium brownii* var. *viridulum* Baker.

Since the therapeutic effects of TCM compounds are generally exerted through gastrointestinal absorption, particularly for gastrointestinal diseases, oral and rectal administration offer significant advantages [18]. Therefore, based on the characterization of the prototype components of LHHZ, we further investigated the distribution of its chemical constituents in mouse gastrointestinal tissues following intragastric administration using liquid chromatography-mass spectrometry (Figure 1B,C). Analysis of gastric tissues revealed 55 compounds after LHHZ administration (Supplementary Table S1-2). Comparative analysis with the original components of LHHZ indicated that 19 compounds originated from *Coptis chinensis*, 23 from *Pogostemon cablin*, and 15 from *Lilium brownii* var. *Viridulum* Baker. Similarly, 48 compounds were detected in intestinal tissues (Supplementary Table S1-3), comprising 15 components from *Coptis chinensis*, 23 from *Pogostemon cablin*, and 12 from *Lilium brownii* var. *Viridulum* Baker. These results suggest that the active components present and distributed in the gastrointestinal tissues may be closely associated with the pharmacodynamic material basis and mechanisms of action of LHHZ.

### 2.2. LHHZ Ameliorates Gastric Mucosal Damage and Inhibits Inflammatory Response in CAG Mice

**Figure 2:**
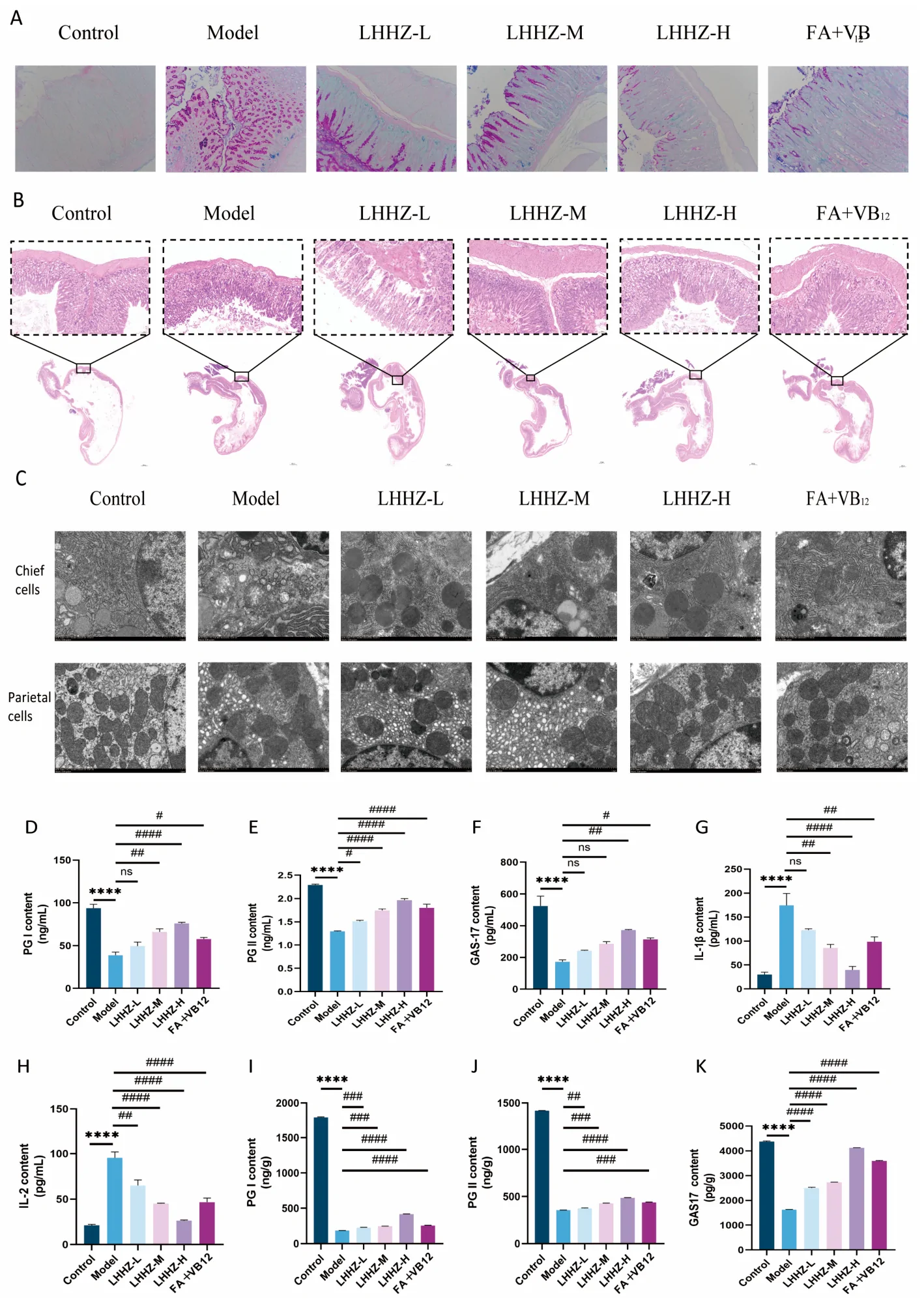
Effects of the Lianhua Huazhuo (LHHZ) formula on gastric tissue histopathology and serum/tissue biochemical indices in chronic atrophic gastritis (CAG) mice. Groups: Control (normal control group), Model (CAG model group), LHHZ-L (low-dose LHHZ treatment group), LHHZ-M (medium-dose LHHZ treatment group), LHHZ-H (high-dose LHHZ treatment group), and FA+VB (positive control group treated with folic acid and vitamin B complex). (**A**) Alcian blue-periodic acid-Schiff (AB-PAS) staining of gastric mucosa (20× magnification) for assessment of mucosal glycoprotein changes. (**B**) Hematoxylin and eosin (HE) staining of gastric tissue (20× magnification) for histopathological evaluation. (**C**) Transmission electron microscopy (TEM) observation of mitochondrial ultrastructure in gastric tissues. (**D**–**F**) Serum levels of pepsinogen I (PG I), pepsinogen II (PG II), and gastrin-17 (GAS-17). (**G**,**H**) Serum levels of interleukin-1β (IL-1β) and interleukin-2 (IL-2). (**I**–**K**) Gastric tissue levels of PG I, PG II, and GAS-17. *n* = 6 per group. **** *p* < 0.0001. Compared with model group ^#^ *p* < 0.05, ^##^ *p* < 0.01, ^###^ *p* < 0.001, ^####^ *p* < 0.0001 vs. model group.

Given that histopathological examination is the diagnostic gold standard for CAG, we first assessed gastric tissue morphology using Alcian Blue-Periodic Acid Schiff (AB-PAS) and Hematoxylin and eosin (H&E) staining. Notably, CAG model mice exhibited substantial pathological alterations, including thinning of the fundic glands, intestinal metaplasia of the gastric antrum, a reduction in the number of parietal cells, and increased mitotic activity. Furthermore, the number of neutral mucus-secreting cells in the gastric mucosa of the model group was elevated, indicative of glands secreting acidic mucus—a characteristic feature of intestinal metaplasia consistent with the pathological changes of CAG (Figure 2A,B). Following LHHZ intervention, gastric mucosal damage was alleviated, and intestinal metaplasia was reduced, suggesting that LHHZ can inhibit the progression of MNU-induced gastric mucosal injury and intestinal metaplasia.

Ultrastructural observation revealed that, compared with the blank control group, nuclei of gastric mucosal cells in the model group were pyknotic, the number of mitochondria in the cytoplasm was reduced with abnormal morphology, cristae were sparse and fragmented, and the rough endoplasmic reticulum was swollen. After intervention with the FA + VB12 group and each LHHZ dose group, nuclei appeared oval or elliptical. Compared with the model group, the number of mitochondria was increased, the proportion of mitochondria with disrupted cristae was reduced, most mitochondria exhibited normal morphology and orderly arrangement, and the rough endoplasmic reticulum was relatively well-organized (Figure 2C). Among the treatment groups, the LHHZ high-dose group exhibited the most pronounced protective effect.

According to the Expert Consensus on the Integrated Traditional Chinese and Western Medicine Diagnosis and Treatment of Chronic Atrophic Gastritis [19], there are two primary approaches for the diagnosis and evaluation of CAG: pathological examination of gastric mucosal specimens, which serves as the diagnostic gold standard, and detection of serum PGI, PGII, and GAS-17, which are serological markers of gastric mucosal atrophy and can be used for screening atrophic gastritis. Therefore, we further measured gastric function indicators in the serum of CAG mice. Results demonstrated that, compared with the control group, serum levels of PGI, PGII, and GAS-17 in the model group were significantly decreased (*p* < 0.05). In contrast, levels of these markers in each LHHZ dose group and the FA + VB12 group were significantly increased relative to the model group (*p* < 0.05) (Figure 2D–F). The trends of PGI, PGII, and GAS-17 levels in gastric tissues were consistent with those observed in serum (Figure 2I–K). To further evaluate the systemic inflammatory status of model mice, serum levels of pro-inflammatory cytokines were measured. The results indicated that IL-1β and IL-2 levels in the model group were significantly elevated (*p* < 0.05), whereas these inflammatory markers were significantly reduced following drug intervention (*p* < 0.05) (Figure 2G,H).

These results indicate that LHHZ alleviates gastric mucosal damage and gastric function decline in CAG mice and ameliorates the MNU-induced increase in inflammatory factor levels.

### 2.3. Potential Biological Targets of LHHZ in CAG Analyzed by Network Pharmacology

**Figure 3:**
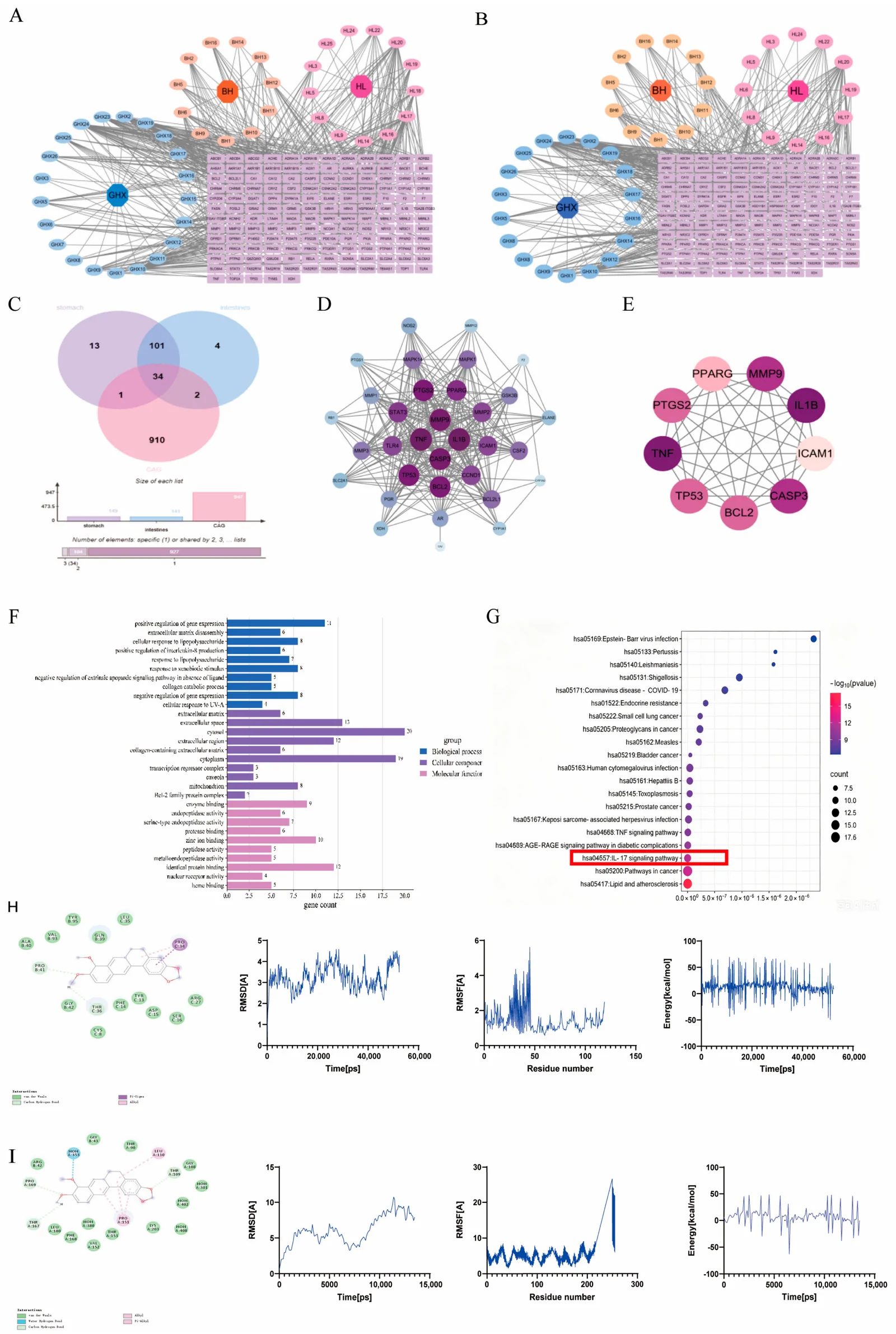
Network pharmacology analysis and molecular dynamics simulation of the Lianhua Huazhuo (LHHZ) formula against chronic atrophic gastritis (CAG). (**A**) Effective component–target interaction network in gastric tissue. (**B**) Effective component–target interaction network in intestinal tissue. HL: *Coptidis Rhizoma*; GHX: *Pogostemonis Herba*; BH: *Lilii Bulbus*. (**C**) Venn diagram showing the intersection between targets of LHHZ effective components and known CAG disease targets. (**D**) Protein–protein interaction (PPI) network of overlapping targets. (**E**) Identification of core hub targets from the PPI network. (**F**) Gene Ontology (GO) enrichment analysis of core targets. (**G**) Kyoto Encyclopedia of Genes and Genomes (KEGG) pathway enrichment analysis of core targets. (**H**) Molecular dynamics simulation of the berberine–interleukin-1β (IL-1β) complex. (**I**) Molecular dynamics simulation of the berberine–tumor necrosis factor-α (TNF-α) complex.

Based on the identified chemical components of LHHZ, a total of 135 potential drug targets were predicted for the components distributed in gastric and intestinal tissues through systematic retrieval from the TCMSP database and the SwissTargetPrediction platform. A total of 947 disease-related targets were obtained by screening the GeneCards database for CAG-associated targets. The intersection between drug and disease targets was analyzed using a bioinformatics online tool, and a Venn diagram was constructed, ultimately identifying 34 common targets (Figure 3A–C). Protein–protein interaction (PPI) information was obtained by importing the intersection targets into the STRING database, yielding 33 nodes and 300 edges. Nine core targets were identified through network centrality analysis (degree, betweenness, closeness), including IL-1β, TNF, CASP3, MMP9, BCL2, TP53, PTGS2, PPARG, and ICAM1 (Figure 3D,E). Gene Ontology (GO) analysis indicated that the main biological processes involved were positive regulation of gene expression, extracellular matrix disassembly, and cellular response to lipopolysaccharide; the primary enriched cellular components were the extracellular matrix, extracellular space, and cytoplasm; and molecular functions were predominantly associated with enzyme binding, endopeptidase activity, and serine-type endopeptidase activity (Figure 3F). Significantly enriched Kyoto Encyclopedia of Genes and Genomes (KEGG) pathways included lipid metabolism and atherosclerosis, cancer-related pathways, the IL-17 signaling pathway, and the AGE-RAGE signaling pathway in diabetic complications (Figure 3G).

Subsequently, molecular docking analysis was performed on the top 20 enriched pathway-related targets ranked by binding affinity. Nine core targets were identified using the CytoNCA plugin, with the top four being TNF-α, IL-1β, CASP3, and MMP9. Docking simulations were conducted between these core targets and their corresponding high-degree chemical components, including berberine, jatrorrhizine, pogonol, colchicine, genkwanin, and phenolic glycerol glucoside. All docking scores were <−7 kcal·mol^–1^, indicating strong binding activity (Supplementary Figure S1A,B), suggesting that these proteins may serve as potential targets of LHHZ against CAG. Given that previous pharmacodynamic studies indicated a potential anti-inflammatory effect of LHHZ, the berberine–IL-1β and berberine–TNF-α complexes were selected for molecular dynamics simulation. RMSD analysis showed that the berberine–IL-1β complex stabilized after 7000 ps, while the berberine–TNF-α complex stabilized after 48,000 ps, indicating relatively stable binding for both complexes. Binding free energy analysis demonstrated that IL-1β and TNF-α could form highly stable complexes with berberine (Figure 3H,I). In summary, the underlying molecular mechanism of LHHZ against CAG may involve the active component berberine targeting proteins such as IL-1β and TNF-α, thereby regulating inflammatory factors and contributing to the ameliorative effects observed in this model.

### 2.4. LHHZ Exerts a Therapeutic Effect on CAG by Regulating the IL-17 Signaling Pathway

As a key pathogenic cytokine and therapeutic target in the body’s defense against autoimmune diseases, inflammatory disorders, and cancer, IL-17 can drive inflammatory responses by activating NF-κB and inducing the expression of genes encoding pro-inflammatory factors and matrix metalloproteinases [20].

**Figure 4:**
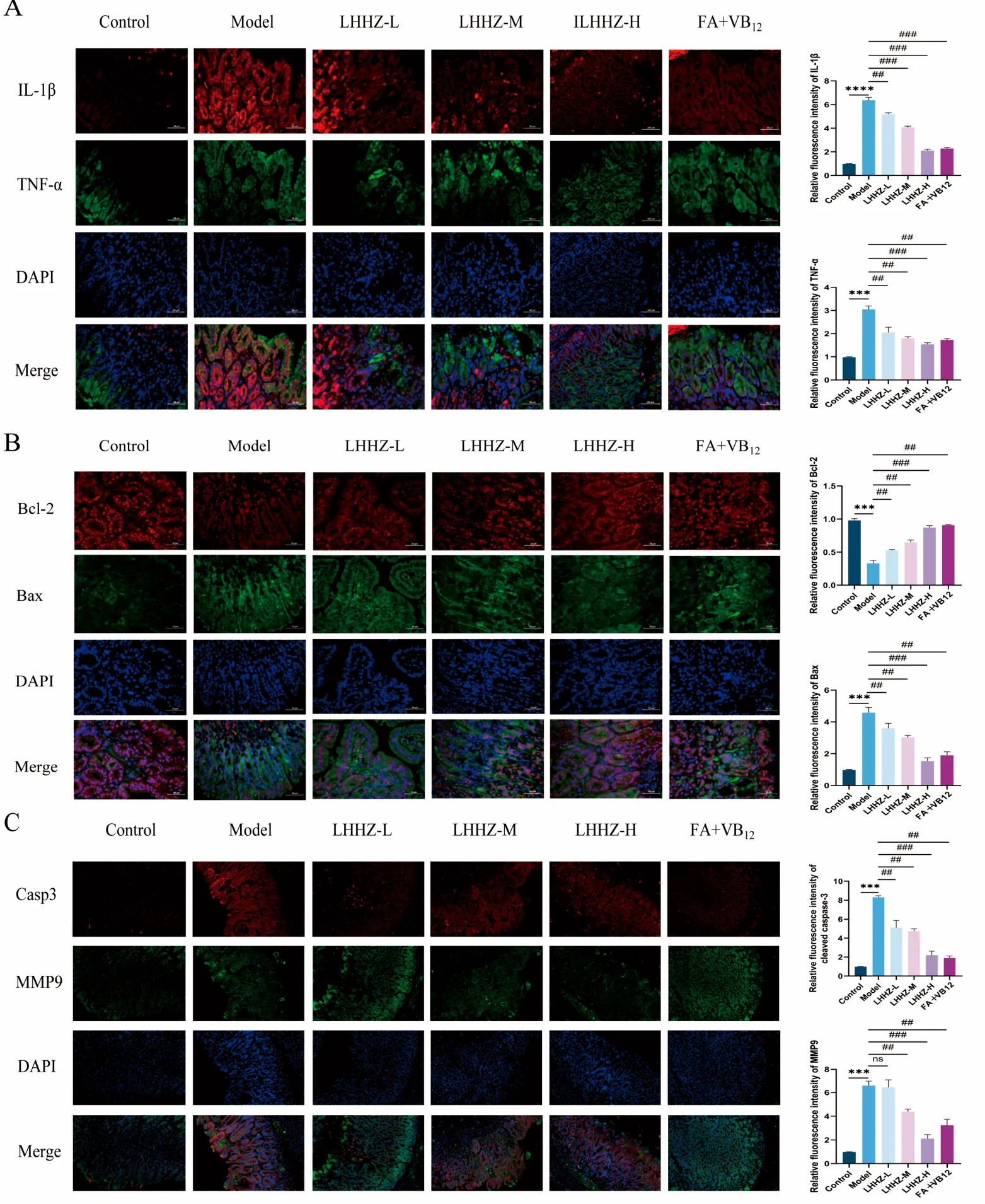
Immunofluorescence staining analysis of protein expression in gastric tissues. (**A**–**C**) Representative immunofluorescence images and quantitative analysis of interleukin-1β (IL-1β), tumor necrosis factor-α (TNF-α), B-cell lymphoma 2 (Bcl-2), Bcl-2-associated X protein (Bax), cleaved caspase-3, and matrix metalloproteinase-9 (MMP-9) in gastric tissue sections (20× magnification). *n* = 3 per group. *** *p* < 0.001, **** *p* < 0.0001. Compared with model group ## *p* < 0.01, ### *p* < 0.001, vs. model group.

Immunofluorescence staining was employed to assess the expression of inflammatory and apoptosis-related proteins in gastric mucosal tissues of mice. The results demonstrated that fluorescence intensities of IL-1β, TNF-α, Bax, Caspase-3, and MMP9 were significantly elevated in the model group, whereas the fluorescence intensity of Bcl-2 was markedly reduced. LHHZ intervention effectively reversed these pathological alterations: compared with the model group, all treatment groups exhibited decreased fluorescence intensity of IL-1β, TNF-α, Bax, Caspase-3, and MMP9, and increased fluorescence intensity of Bcl-2 (*p* < 0.05) (Figure 4A–C).

**Figure 5:**
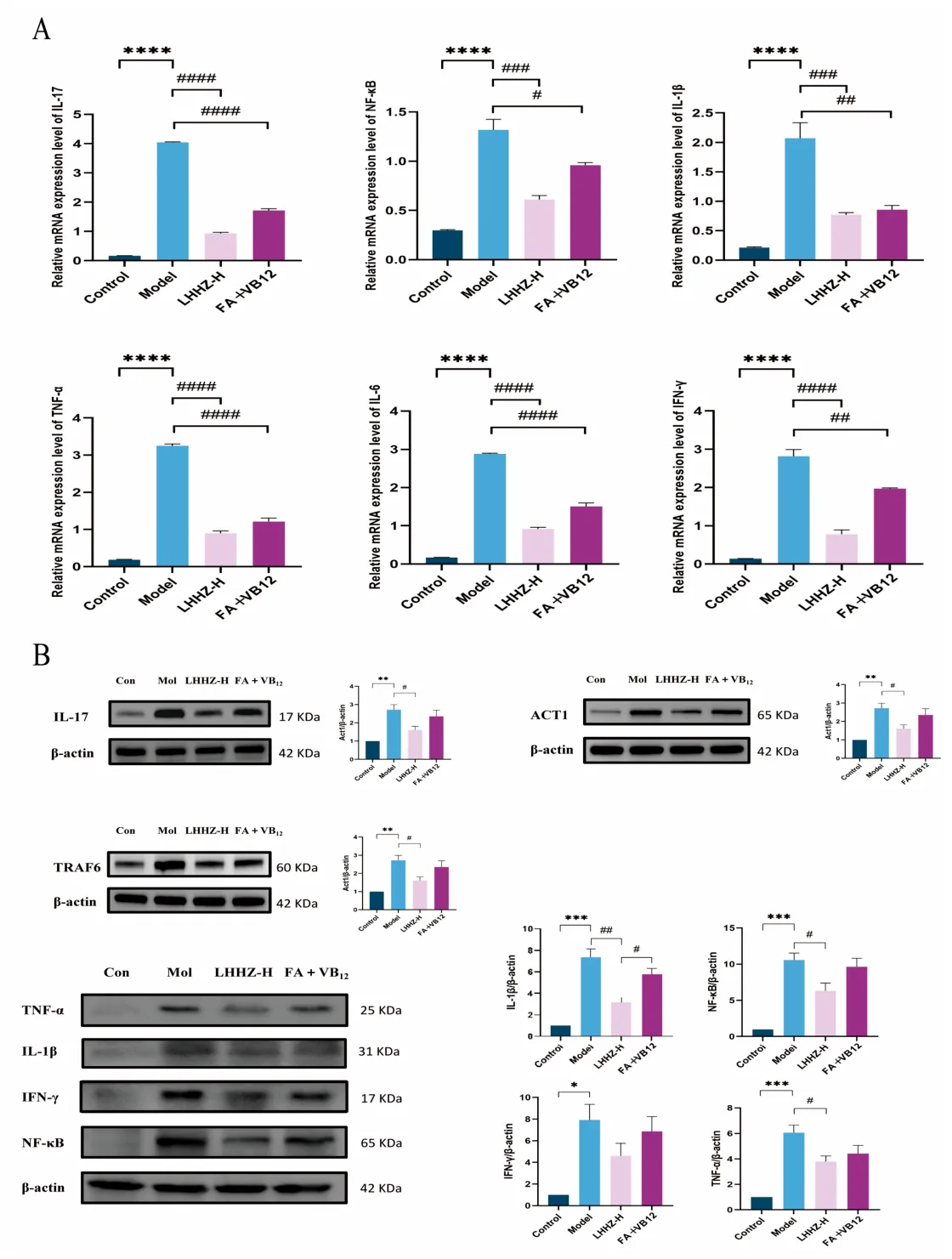
Regulation of the interleukin-17 (IL-17) signaling pathway by the Lianhua Huazhuo (LHHZ) formula in gastric tissues. (**A**) Relative messenger ribonucleic acid (mRNA) expression levels of interleukin-17A (IL-17A), tumor necrosis factor-α (TNF-α), interleukin-1β (IL-1β), interferon-γ (IFN-γ), and nuclear factor-κB (NF-κB) determined by quantitative real-time polymerase chain reaction (qRT-PCR). *n* = 3 per group. (**B**) Protein expression levels of IL-17A, adaptor protein Act1, TNF receptor-associated factor 6 (TRAF6), TNF-α, IL-1β, IFN-γ, and NF-κB detected by Western blotting (WB). Con: control group; Mol: model group. * *p* < 0.05, ** *p* < 0.01, *** *p* < 0.001, **** *p* < 0.0001; Compared with model group ^#^ *p* < 0.05, ^##^ *p* < 0.01, ^###^ *p* < 0.001, ^####^ *p* < 0.0001 vs. model group.

To investigate whether LHHZ modulates the IL-17 signaling pathway and its downstream targets, we measured gene and protein expression and localization in each group of mice. Given the dose-dependent effects observed in prior efficacy assessments and the notable protective effect of the high-dose group, the LHHZ high-dose group was selected for this analysis. At the gene level, mRNA expression levels of IL-17A, TNF-α, IL-1β, IL-6, NF-κB, and IFN-γ in the gastric tissues of the model group were significantly elevated (*p* < 0.05), indicating pronounced transcriptional activation of the IL-17 signaling pathway and associated inflammatory genes in CAG mice. In contrast, the mRNA levels of these genes were significantly reduced in the treatment groups compared with the model group (*p* < 0.05) (Figure 5A). At the protein level, expression trends of IL-17A, Act1, TRAF6, NF-κB, TNF-α, IL-1β, and IFN-γ mirrored the mRNA results, and all were significantly decreased in the treatment groups relative to the model group (*p* < 0.05) (Figure 5B). These results further indicate that LHHZ can regulate IL-17 signaling pathway expression at the protein level, thereby contributing to the amelioration of gastric pathology in this mouse model of CAG.

### 2.5. LHHZ Exerts a Protective Effect on CAG via the Gut Microbiota-SCFAs Axis

GM plays a pivotal role in human health and modulates a variety of pathophysiological processes. Certain microorganisms are capable of maintaining intestinal homeostasis, alleviating inflammation, supporting neural function, and regulating metabolic activity. SCFAs are a class of fatty acids produced through the fermentation of indigestible polysaccharides by GM [21]. Accumulating evidence indicates that GM and SCFAs interact via multiple pathways, with key mechanisms involving inflammation and immune responses, intestinal barrier integrity, the gut–brain axis, and metabolic regulation [22]. Based on the expression patterns of pro-inflammatory cytokines in CAG mice observed in our previous experiments, we hypothesized that these phenomena may be closely linked to the GM–SCFA axis. Accordingly, to test this hypothesis, we analyzed the GM composition and SCFA levels in intestinal samples from CAG mice.

**Figure 6:**
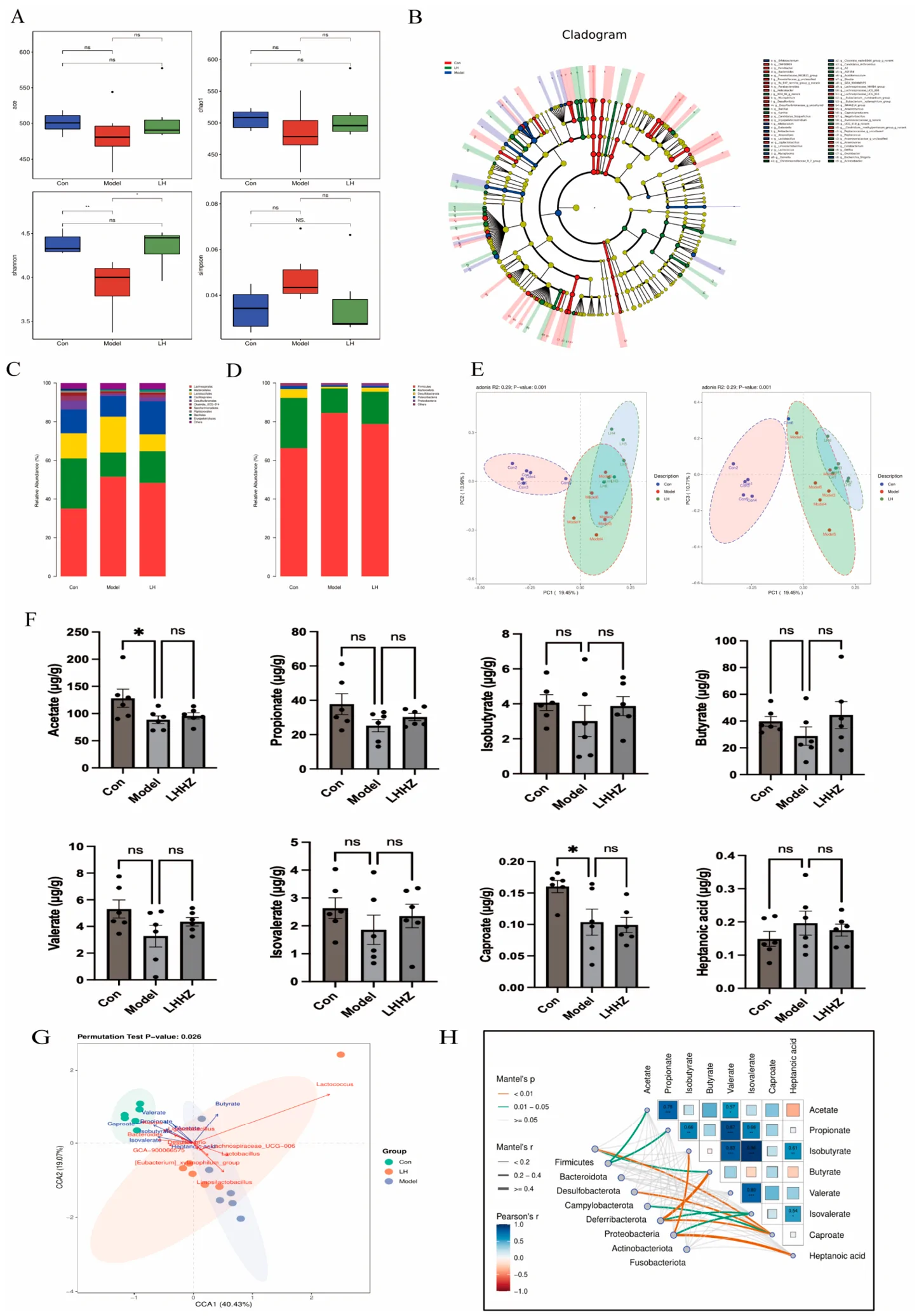
Integrated correlation analysis of gut microbiome and metabolome in experimental groups. (**A**) Alpha diversity indices (ACE, Chao1, Shannon, and Simpson) of gut microbiota across groups. (**B**) Linear discriminant analysis effect size (LEfSe) cladogram showing phylogenetic distribution of differentially abundant microbiota among groups. (**C**) Relative abundance of gut microbiota at the phylum level. (**D**) Relative abundance of gut microbiota at the order level. (**E**) Beta diversity analysis by weighted UniFrac principal coordinate analysis (PCoA) of genus-level microbiota. (**F**) Intestinal short-chain fatty acid (SCFA) concentrations in each group. (**G**) Canonical correspondence analysis (CCA) illustrating relationships between microbiota, metabolites, and experimental groups. (**H**) Mantel test results for correlations between microbiome and metabolome matrices. Con: control group; Model: model group; LH: LHHZ-treated group. * *p* < 0.05, ** *p* < 0.01, *** *p* < 0.001 vs. model group.

16S rRNA sequencing revealed that intestinal contents comprised 13 phyla, 19 classes, 51 orders, 98 families, 208 genera, 329 species, and 767 OTUs. Alpha diversity analysis indicated that the Shannon index was significantly lower in the model group compared with the control group (*p* < 0.05), whereas the Shannon index in the LHHZ treatment group was significantly higher than that in the model group (*p* < 0.05). No significant differences were observed among groups for the ACE index, Chao1 index, or Simpson index (Figure 6A). Principal coordinates analysis (PCoA) of beta diversity showed that the first three principal coordinates (PC1 = 19.45%, PC2 = 13.96%, PC3 = 10.71%) accounted for approximately 44% of the variation in the microbial community, indicating structured differences within the dataset (Figure 6E). These results suggest that LHHZ can effectively enhance both the diversity and abundance of GM.

Analysis of differences in fecal microbiota composition revealed that LEfSe analysis identified a total of 108 differentially abundant taxa across classification levels, including 53 in the control group, 20 in the model group, and 35 in the LHHZ group (Supplementary Figure S2). The cladogram showed that the control group was significantly enriched with taxa such as Spirochaeta of the phylum Deferribacteres; compared with the control group, the intestines of CAG model mice exhibited increased abundance of taxa such as Bifidobacterium of the phylum Actinobacteria; the LHHZ group was enriched with taxa such as ASF356 of the phylum Bacteroidetes (Figure 6B). Significant differences were observed in dominant phyla among the three groups. The abundance of Firmicutes in the model group was significantly higher than that in the control group, whereas it was reduced in the LHHZ group relative to the model group. Bacteroidetes were enriched in the control group, decreased in the model group, and partially restored following LHHZ administration. The Firmicutes/Bacteroidetes (F/B) ratio was significantly elevated in the CAG model, and LHHZ significantly decreased this ratio. At the order level, abundances of Lachnospirales and Lactobacillales in the model group were significantly higher than those in the control group, whereas abundances of Bacteroidales and Oscillospirales were significantly lower. LHHZ treatment ameliorated these alterations in GM composition. Notably, Bacteroidales and Oscillospirales are major producers of SCFAs, including butyric acid and acetic acid (Figure 6C,D).

Targeted metabolomics analysis detected eight SCFAs in the intestines of CAG mice: acetic acid, propionic acid, isobutyric acid, butyric acid, valeric acid, isovaleric acid, caproic acid, and heptanoic acid. Among these SCFAs, heptanoic acid exhibited an increasing trend in the model group, whereas the other seven SCFAs showed a decreasing trend. LHHZ intervention normalized the concentrations of all eight SCFAs (Figure 6F), suggesting that the protective effects of LHHZ observed in this CAG model may be associated with these metabolites.

To further elucidate the relationship between SCFAs and GM, canonical correspondence analysis (CCA) was performed. Results demonstrated significant co-variation between environmental factors and microbial composition (CCA1 = 40.43%, CCA2 = 19.07%, total explained variance = 59.47%, *p* = 0.026), indicating that metabolic factors drive substantial shifts in microbiota composition (Figure 6G). Heatmap analysis included the 46 OTUs with the most significant differential abundances (annotated at the genus level) and all eight SCFAs (Supplementary Figure S3). Mantel test analysis further quantified associations between individual metabolites and the phylum-level community matrix. These analyses revealed that at the genus level, Ligilactobacillus, Blautia, Bacteroides, GCA-900066575, and Desulfovibrio exhibited strong correlations with seven SCFAs (excluding butyric acid), whereas Lactococcus was specifically correlated with butyric acid (Figure 6H). These results suggest that alterations in GM composition are closely linked to SCFA metabolism in CAG. These data suggest that the protective effects of LHHZ on gastric mucosal damage and inflammatory infiltration in this mouse model may be associated with modulation of the GM-SCFA axis.

## 3. Discussion

CAG is a prevalent gastrointestinal disorder characterized by progressive gastric mucosal injury and glandular atrophy and is widely recognized as a critical precancerous condition of gastric cancer. Because CAG is closely associated with persistent mucosal inflammation, epithelial damage, and intestinal metaplasia, effective management should not only mitigate inflammation but also promote mucosal repair and delay disease progression. Given the increasing global burden of CAG, the development of effective and well-tolerated therapeutic strategies remains a clinically significant challenge [23].

The pathogenesis of CAG is complex and multifactorial. Chronic exposure to *Helicobacter pylori*, dietary irritants, immune dysregulation, and bile or duodenal reflux contributes to sustained gastric mucosal inflammation, progressively disrupting epithelial integrity and glandular architecture. In this process, infiltrating inflammatory cells release cytokines such as TNF-α, IL-1β, and IL-6, thereby amplifying local immune responses, impairing mucosal barrier function, and interfering with tissue repair [12]. Accordingly, suppression of chronic inflammation and restoration of mucosal homeostasis are central objectives in CAG treatment.

Current conventional therapies for CAG demonstrate limited capacity to reverse established mucosal atrophy, and some interventions are associated with suboptimal efficacy or adverse effects [5,24]. In contrast, TCM, characterized by multi-component and multi-target mechanisms, may offer advantages in managing complex chronic disorders such as CAG. Recent clinical studies and systematic evaluations of TCM-based interventions for CAG have suggested beneficial effects on symptom relief, endoscopic or histopathological improvement, and inflammatory regulation [11,12]. Currently, no high-quality randomized controlled trials have specifically evaluated LHHZ, and its clinical efficacy has not been established. These findings from TCM-based studies nevertheless provide a clinical context for the present preclinical investigation.

In the present study, LHHZ demonstrated significant protective effects in CAG mice. Histopathological and ultrastructural analyses revealed that LHHZ alleviated gastric mucosal injury, reduced intestinal metaplasia, and improved the ultrastructural integrity of gastric mucosal cells. Concurrently, LHHZ restored gastric functional markers, including PGI, PGII, and GAS-17, and decreased levels of pro-inflammatory mediators such as IL-1β and IL-2 in serum and gastric tissues. These results indicate that LHHZ not only ameliorates tissue morphology but also promotes functional recovery of the injured gastric mucosa. The pharmacodynamic findings support a protective role of LHHZ against CAG-associated gastric injury, primarily through attenuation of inflammation and improvement of the mucosal microenvironment.

To elucidate the material basis underlying these effects, UHPLC-Q-Orbitrap HRMS was employed to characterize the chemical constituents of LHHZ and the absorbed components in target tissues. A total of 85 potential bioactive compounds were identified in the formula, among which 55 and 48 absorbed components were detected in gastric and intestinal tissues, respectively. These tissue-distributed constituents were primarily derived from *Coptis chinensis*, *Pogostemon cablin*, and *Lilium brownii* var. *Viridulum* Baker, suggesting that these three herbs constitute the principal pharmacodynamic basis of LHHZ. This inference aligns with previous reports indicating that *Coptis chinensis* exhibits anti-inflammatory and microbiota-modulating activities, *Pogostemon cablin* exerts protective effects on gastrointestinal inflammation and motility, and *Lilium brownii* var. *viridulum* Baker possesses immunomodulatory and anti-inflammatory properties [25,26,27,28,29]. Therefore, protective effects of LHHZ observed in this study are likely attributable to synergistic actions among its core herbal components.

To further delineate the mechanistic basis of LHHZ, we integrated network pharmacology, molecular docking, molecular dynamics simulation, and experimental validation. Based on the absorbed components identified in gastrointestinal tissues, 135 putative drug targets were screened, and 34 core targets were obtained by intersection with CAG-related targets. PPI analysis revealed several hub targets, including IL-1β, TNF, and CASP3, while GO and KEGG analyses indicated significant enrichment in inflammation-related biological processes and signaling pathways, particularly the IL-17 signaling pathway. Molecular docking further demonstrated that key active compounds, such as berberine and jatrorrhizine, exhibited strong binding affinities for core inflammatory targets including IL-1β and TNF-α, and molecular dynamics simulations confirmed the stability of these ligand–target complexes. These findings suggest that the absorbed bioactive constituents of LHHZ may exert direct regulatory effects on pivotal inflammatory nodes involved in CAG progression.

**Figure 7:**
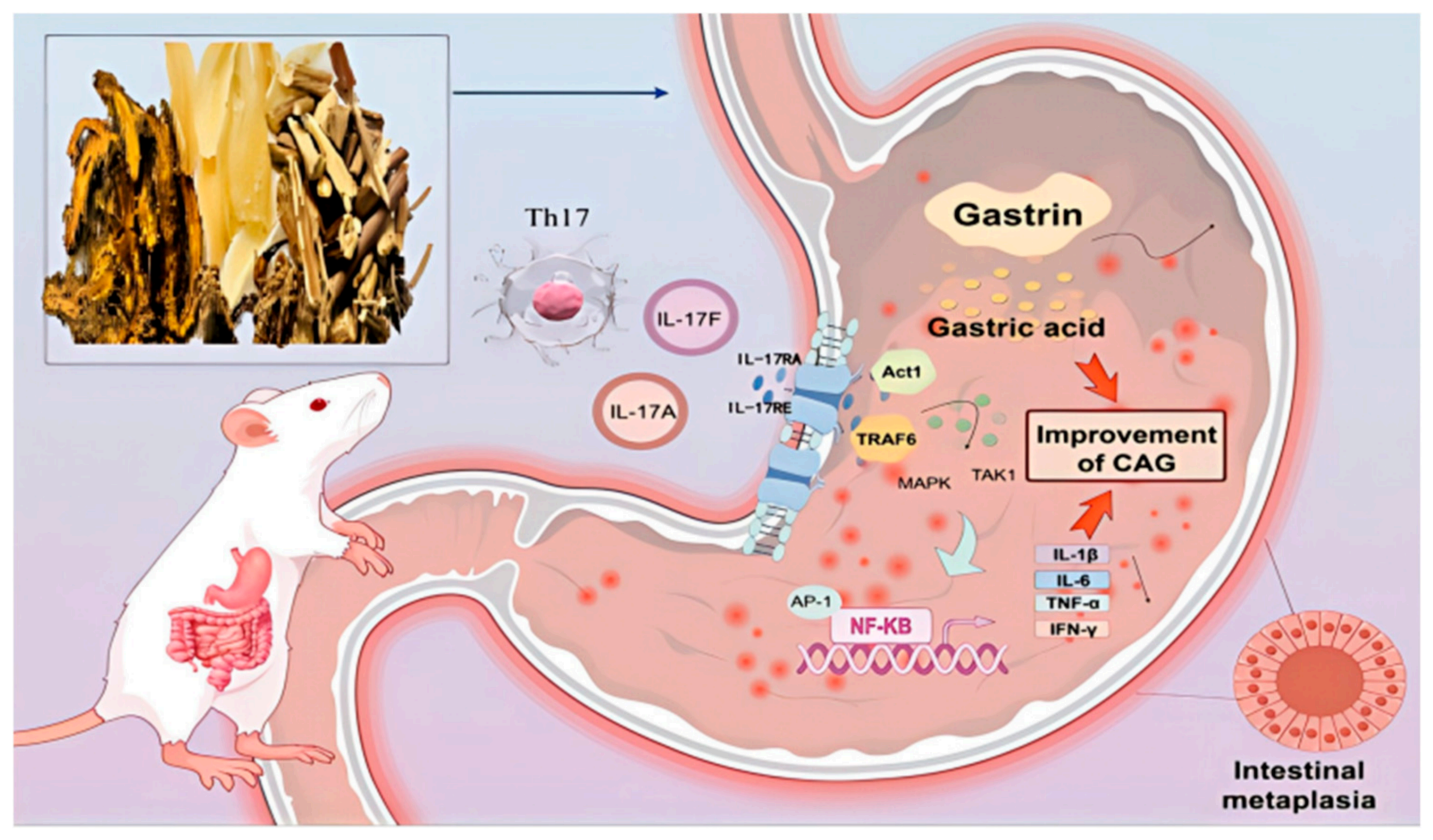
Schematic mechanism diagram illustrating the protective effect of the Lianhua Huazhuo (LHHZ) formula against chronic atrophic gastritis (CAG) via modulation of the interleukin-17 (IL-17) signaling pathway, gut microbiota, and metabolic homeostasis.

Experimental validation further supported these predictions. LHHZ significantly downregulated the expression of IL-17A, Act1, TRAF6, and NF-κB in gastric tissues of CAG mice, as demonstrated by immunofluorescence, RT-qPCR, and Western blot analyses. IL-17 is a key mediator of chronic inflammatory responses and can activate downstream NF-κB signaling, thereby promoting the expression of pro-inflammatory factors such as IL-1β and TNF-α [20,30]. Act1 serves as an essential adaptor in IL-17 signaling and mediates recruitment of TRAF6, which subsequently activates downstream inflammatory cascades [30,31,32,33]. In this context, suppression of the IL-17/Act1/TRAF6/NF-κB axis by LHHZ offers a plausible mechanistic link that may contribute to the observed reductions in IL-1β, IL-6, TNF-α, and IFN-γ, and to the alleviation of gastric mucosal inflammation in this model (Figure 7). Together with the results of network pharmacology and molecular interaction analyses, these data support a model in which representative compounds of LHHZ, particularly berberine-related alkaloids, target core inflammatory mediators and disrupt pathological IL-17-driven signaling, thereby preserving gastric mucosal structure and function.

An additional strength of this study is its integration of anti-inflammatory regulation in the stomach with modulation of intestinal microecology. Increasing evidence indicates that GM participates in upper gastrointestinal disorders via immune, metabolic, and barrier-related mechanisms. In the present study, CAG was associated with marked gut microbial dysbiosis, characterized by decreased α-diversity, altered β-diversity, an increased F/B ratio, enrichment of Lachnospirales and Lactobacillales, and depletion of Bacteroidales and Oscillospirales. As Bacteroidales and Oscillospirales are major SCFA-producing taxa, these alterations suggest a disrupted metabolic environment in CAG. Consistently, targeted metabolomics revealed abnormal levels of eight SCFAs in model mice, indicating that CAG-associated microbial imbalance was accompanied by impaired microbial metabolite homeostasis.

Notably, LHHZ substantially ameliorated these abnormalities. Treatment increased microbial α-diversity, normalized the F/B ratio, restored SCFA-producing bacteria, and rebalanced the concentrations of all eight measured SCFAs. Moreover, CCA and Mantel test analyses demonstrated significant associations between microbial composition and SCFA profiles, supporting a functional link between microbiota remodeling and metabolic recovery. Given the established roles of SCFAs in maintaining epithelial energy supply, enhancing mucosal barrier integrity, regulating luminal pH, and modulating immune responses [34,35,36], these preclinical findings suggest that LHHZ may mitigate CAG-related gastric pathology in this mouse model not only through direct suppression of gastric inflammation but also via restoration of gut microbial ecology and metabolite-mediated host protection. This gut–stomach regulatory axis may represent an important complementary mechanism underlying the protective effects of LHHZ in this model.

The role of IL-17 warrants cautious interpretation. Although IL-17 is a recognized pro-inflammatory mediator in pathological contexts, it has also been implicated in maintenance of mucosal defense and intercellular barrier integrity in the intestine under specific physiological or disease conditions. Therefore, a theoretical concern is whether inhibition of IL-17 signaling by LHHZ might negatively impact intestinal homeostasis. Our current data do not indicate such a detrimental effect. On the contrary, LHHZ improved gut microbial diversity, corrected microbial structural imbalance, restored SCFA-producing bacteria, and normalized SCFA metabolism, changes that are generally associated with enhanced barrier function and reduced inflammatory susceptibility. Thus, within the present experimental setting, LHHZ appears to modulate excessive or pathological IL-17-mediated inflammation rather than indiscriminately suppress protective mucosal immunity. Nevertheless, because intestinal tight junction proteins, epithelial permeability, and immune-cell subsets were not directly assessed, this issue warrants further dedicated investigation.

Several limitations of this study should be acknowledged. First, high-quality randomized controlled trials specifically evaluating LHHZ in CAG are currently lacking; therefore, the present findings should be interpreted as preclinical evidence rather than direct clinical confirmation. Second, although our results consistently indicate involvement of the IL-17/Act1/TRAF6/NF-κB pathway, causal validation using pharmacological inhibitors, genetic interference, or gain-/loss-of-function approaches was not performed. Third, the present study was conducted in a mouse model with a relatively limited sample size, which may restrict the statistical power and generalizability of the findings. Larger-scale animal studies are needed to further strengthen the interpretation and translational relevance of the observed effects. Fourth, immune-cell-level evidence remains limited: gastric or intestinal T-cell subsets, including Th17 cells, were not isolated, and flow cytometric analysis was not conducted to directly confirm the immunoregulatory effects of LHHZ. Fifth, given the context-dependent protective role of IL-17 in intestinal mucosal biology, direct assessment of intestinal barrier markers and inflammatory status is needed to further evaluate the safety and tissue specificity of IL-17 modulation by LHHZ. Sixth, the LHHZ decoction used in this study was prepared from a single batch, and inter-batch consistency was not assessed. Finally, no direct comparison was made between LHHZ and the prototype formula XLHZ, limiting the ability to fully evaluate the scientific implications of formula simplification and optimization. Future studies addressing these issues, together with clinical validation of LHHZ, will be important for strengthening the mechanistic and translational basis for its further investigation in CAG.

Overall, in a mouse model of CAG, LHHZ exerted protective effects via a multi-level mechanism involving improvement of gastric mucosal pathology and function, inhibition of IL-17/Act1/TRAF6/NF-κB-mediated inflammatory signaling, and restoration of GM–SCFA homeostasis. These preclinical findings support LHHZ as a promising candidate for further investigation in CAG and offer a mechanistic framework for its continued preclinical development. Nevertheless, further larger-scale animal studies and well-designed clinical trials will be required to confirm these observations and evaluate their translational relevance.

## 4. Materials and Methods

### 4.1. Experimental Materials and Ethical Statement

#### 4.1.1. Drugs and Reagents

*Coptis chinensis* Franch., *Pogostemon cablin* (Blanco) Benth., and *Lilium brownii* var *Viridulum* Baker. were obtained from Sinopharm Lerentang Pharmaceutical Co., Ltd. (Shijiazhuang, China). The plant species were authenticated using the World Flora Online database (http://www.worldfloraonline.org) on 17 October 2025. All medicinal materials were further authenticated by Professor Zhang Yixin, Dean of the School of Pharmacy, Hebei University of Chinese Medicine. N-Methyl-N-nitrosourea (MNU; Cat. No.: K2320260) was purchased from Aladdin Biochemical Technology Co., Ltd. (Shanghai, China). Vitamin B12 (Cat. No.: V820400) and folic acid (Cat. No.: F809519) were acquired from Macklin Biochemical Technology Co., Ltd. (Shanghai, China).

#### 4.1.2. Software

Network pharmacology target prediction and visualization were performed via Cytoscape (Version 3.9.1, National Institutes of Health, Bethesda, MD, USA). Molecular docking simulations were performed using the CB-Dock2 online tool. A 100 ns molecular dynamics simulation of ligand–receptor complexes was carried out with YASARA (Version 10.3.16, YASARA Biosciences GmbH, Vienna, Austria). Raw 16S rRNA gene sequencing data were processed and analyzed using QIIME2 (Version 2023.5, University of Colorado Boulder, Boulder, MD, USA). Statistical analysis was performed with SPSS (Version 27.0, IBM, Armonk, NY, USA) and GraphPad Prism (Version 8.0.2, GraphPad Software, San Diego, CA, USA). Correlation heatmaps and all other visualization figures were drawn using R software (Version 4.2.1, R Core Team, Vienna, Austria) with the ggplot2 package.

#### 4.1.3. Experimental Animals and Ethics

Six-week-old SPF male BALB/c mice were obtained from Beijing SPF Biotechnology Co., Ltd. (Beijing, China; License No.: SCXK [Beijing] 2024-0001). The mice were maintained under standardized conditions and allowed a one-week acclimatization period prior to experimentation. All procedures conformed to the Guidelines for the Welfare and Ethical Review of Laboratory Animals of Hebei University of Chinese Medicine (Registration No.: DWLL202305004, date of approval 4 May 2023).

### 4.2. Identification of LHHZ Components

#### 4.2.1. Identification of Gastrointestinal Absorbed Components of LHHZ (UHPLC-Q-Orbitrap HRMS)

##### Chromatographic Conditions

Chromatographic separation was performed on a Vanquish Flex UHPLC system. The mobile phase consisted of 0.1% formic acid aqueous solution (A) and acetonitrile (B). The injection volume was 6.0 μL at a flow rate of 300 nL/min. The gradient elution was set as follows: 0–1 min, 98% A; 1–14 min, 98% A to 70% A; 14–25 min, 70% A to 0% A; 25–28 min, 0% A; 28.1–30 min, back to 98% A.

##### Mass Spectrometric Conditions

Mass spectrometry analysis was carried out on a Q Exactive™ mass spectrometer with a Captive Spray ion source(Thermo Fisher Scientific, Waltham, MA, USA). Data were acquired in full scan/dd-MS^2^ mode within the mass range of 100–1500 *m*/*z*. The resolution was 70,000 for full scan and 17,500 for dd-MS^2^, and the collision energies were set at 10 V, 30 V and 60 V.

##### Sample Preparation

For LHHZ sample preparation, 1.0 g of accurately weighed LHHZ was placed into a 50 mL polypropylene centrifuge tube, and 40 mL of 80% methanol (*v*/*v*) was added for ultrasonic-assisted extraction at 40 kHz for 30 min at ambient temperature. The supernatant was collected and centrifuged at 12,000 rpm for 10 min at 4 °C, and 100 μL was reserved for subsequent analysis. For mouse gastrointestinal tissue samples, 50 mg of tissue was placed into a 2 mL homogenization tube, combined with 100 μL of 80% methanol aqueous solution, and homogenized for 1 min. An additional 900 μL of 80% methanol aqueous solution was added, and the mixture was vortexed for 1 h, followed by centrifugation under the same conditions. Finally, 80 μL of the supernatant was collected for analysis.

### 4.3. Efficacy Evaluation: Establishment of CAG Model and Efficacy Detection

#### 4.3.1. Animal Model Establishment and Administration

##### Grouping and Modeling

After one week of adaptive feeding, a total of 60 SPF-grade BALB/c mice were randomly assigned to six groups (*n* = 10 per group) using a random number table: normal control group, CAG model group, positive control group (folic acid 10.9 mg/kg + vitamin B12 13.6 mg/kg), LHHZ low-dose group (4 g/kg/day, equivalent to the adult clinical dose), LHHZ medium-dose group (8 g/kg/day, twice the adult clinical dose), and LHHZ high-dose group (16 g/kg/day, four times the adult clinical dose). Human equivalent doses were calculated according to the cross-species dose conversion table in Pharmacological Experiment Methodology [37]. Mice in the normal control group were maintained under standard feeding conditions, provided with normal drinking water, and received the same volume of normal saline by gavage. All other groups, except the normal control group, were administered 300 ppm MNU in drinking water, combined with weekly intragastric gavage of 0.2 mL MNU solution (5 mg/3 mL), for 14 consecutive weeks to induce the CAG model [38].

##### Administration Protocol and Efficacy Evaluation

Intervention commenced from the ninth week of modeling, corresponding to the ninth week of the 14-week CAG induction period. The positive control group received intragastric gavage of vitamin B12 (13.6 mg/kg) and folic acid (10.9 mg/kg), while each LHHZ dose group received intragastric administration of the respective drug solution once daily for six consecutive weeks. The normal control group and model group were administered an equivalent volume of normal saline over the same period. At the end of the experiment, mice were fasted for 12 h, anesthetized with pentobarbital sodium, and euthanized by cervical dislocation. Blood samples were collected, and the stomach was immediately excised under sterile conditions. After removal of gastric contents, tissues were gently rinsed with cold normal saline. Gastric tissues were then allocated for different analyses: one portion was fixed in 4% paraformaldehyde for histological staining and immunofluorescence, one portion was fixed in 2.5% glutaraldehyde for transmission electron microscopy, and the remaining tissues were stored at −80 °C for biochemical assays, real-time quantitative polymerase chain reaction, and Western blot analysis. Therapeutic efficacy was assessed by histopathological staining, ultrastructural observation, and measurement of serum and gastric tissue-related indicators. Successful model establishment was confirmed by gastric histopathological alterations, including inflammatory cell infiltration, glandular atrophy, epithelial injury, and intestinal metaplasia, along with changes in gastric functional markers and inflammatory cytokines.

#### 4.3.2. Detection of Gastric Mucosal Pathological Morphology

Gastric tissues were fixed, embedded, and sectioned into paraffin slices. H&E staining was performed to examine mucosal morphology and inflammatory infiltration, whereas AB-PAS staining was used to assess mucus secretion and intestinal metaplasia. Separately, additional tissue samples were fixed, dehydrated, and embedded to prepare ultra-thin sections. Following double staining with uranyl acetate and lead citrate, cellular ultrastructure was observed using a Hitachi H-7650 transmission electron microscope (Hitachi High-Technologies Corporation, Tokyo, Japan).

#### 4.3.3. Detection of Gastric Function Markers

##### Serum Indicators

Serum concentrations of PGI, PGII, and GAS-17 were determined using a Mindray BS-120 automatic biochemical analyzer (Shenzhen Mindray Bio-Medical Electronics Co., Ltd., Shenzhen, China).

##### Tissue Indicators

The contents of PGI, PGII, and GAS-17 in gastric tissues were measured using enzyme-linked immunosorbent assay (ELISA) kits, following the manufacturer’s instructions. Optical density (OD) values were detected with a microplate reader, and concentrations were calculated using standard curves.

### 4.4. Target and Pathway Prediction

#### 4.4.1. Network Pharmacology Target Screening

The gastrointestinal absorbed components of LHHZ and disease-related targets of CAG were analyzed to identify their intersections. Following the screening of core targets, KEGG enrichment analysis was conducted, with particular focus on targets associated with the IL-17 signaling pathway.

#### 4.4.2. Molecular Docking and Molecular Dynamics Simulation

##### Molecular Docking

Based on network pharmacology results, six LHHZ components with high abundance, potential pharmacological activity, and significant target enrichment—berberine, jatrorrhizine, pogonol, colchicine, genkwanin, and phenolic glycerol glucoside—were selected as ligand molecules. The corresponding target proteins included TNF-α, IL-1β, Caspase-3, and MMP9. Two-dimensional (2D) structures of the ligands in SDF format were obtained from the PubChem database, whereas the PDB files of the receptor proteins were retrieved from the RCSB PDB database. Molecular docking simulations were performed using the CB-Dock2 online tool, with a Vina score of <−5 kJ/mol considered indicative of favorable binding affinity between ligands and receptors.

##### Molecular Dynamics Simulation

A 100 ns molecular dynamics simulation of the docked complexes was conducted using YASARA 10.3.16 software. The protonation states were optimized at pH 7.4, and periodic boundary conditions were applied. Following energy minimization and NVT/NPT equilibration, backbone root mean square deviation (RMSD), residue root mean square fluctuation (RMSF), and binding free energy were analyzed to assess the binding stability of the component–target complexes.

### 4.5. Mechanism Verification: Detection of IL-17 Signaling Pathway

#### 4.5.1. Immunofluorescence Detection

Immunofluorescence staining was performed to evaluate the cellular localization and expression levels of IL-1β, TNF-α, Bax, Caspase-3, and MMP9 in gastric tissues. Briefly, gastric tissue sections were fixed, embedded, sectioned, and subjected to antigen retrieval. After blocking with an appropriate blocking solution, the sections were incubated overnight at 4 °C with primary antibodies against IL-1β (Proteintech, Wuhan, China; Cat. No. 16806; 1:250), TNF-α (Servicebio, Wuhan, China; Cat. No. GB11188; 1:500), Bax (Proteintech, Wuhan, China; Cat. No. 50599; 1:2500), Caspase-3 (Servicebio, Wuhan, China; Cat. No. TBG0355; 1:5000), and MMP9 (Zenbio, Chengdu, China; Cat. No. 380831; 1:75). After washing, the sections were incubated with fluorescence-labeled secondary antibodies under dark conditions. Cell nuclei were counterstained with DAPI. Fluorescence images were captured using a fluorescence microscope under identical exposure settings for all groups, and fluorescence intensity was quantified using ImageJ software (Version 1.54f).

#### 4.5.2. RT-qPCR Detection of Gene Transcription Levels

Total RNA was extracted from gastric tissues using TRIzol reagent according to the manufacturer’s instructions. RNA concentration and purity were assessed prior to reverse transcription. Complementary DNA (cDNA) was synthesized using a reverse transcription kit under standard reaction conditions. Quantitative real-time PCR was performed using a SYBR Green-based PCR Master Mix on a real-time PCR system. β-Actin served as the internal reference gene, and transcription levels of IL-17A, TNF-α, IL-1β, IL-6, NF-κB, and IFN-γ were quantified. Relative mRNA expression levels were calculated using the 2^−ΔΔCt^ method. Primer sequences are provided in Supplementary Table S2.

#### 4.5.3. Western Blotting (WB) Detection of Protein Expression Levels

Total proteins were extracted from gastric tissues using RIPA lysis buffer supplemented with protease and phosphatase inhibitors on ice. The lysates were centrifuged, and supernatants were collected for protein analysis. Protein concentrations were determined using a BCA protein assay kit. Equal amounts of protein were mixed with loading buffer, denatured, separated by SDS-PAGE, and transferred onto PVDF membranes. After blocking with 5% non-fat milk at room temperature, membranes were incubated overnight at 4 °C with primary antibodies against IL-17A (Servicebio, Wuhan, China; Cat. No. GB11110; 1:750), Act1 (Proteintech, Wuhan, China; Cat. No. 26692; 1:1000), TRAF6 (Servicebio, Wuhan, China; Cat. No. GB112201; 1:750), NF-κB (Proteintech, Wuhan, China; Cat. No. 10745; 1:2000), TNF-α (Servicebio, Wuhan, China; Cat. No. GB11188; 1:300), IL-1β (Proteintech, Wuhan, China; Cat. No. 16806; 1:6000), IFN-γ (Proteintech, Wuhan, China; Cat. No. 29788; 1:1000), and β-actin (Servicebio, Wuhan, China; Cat. No. GB11001; 1:1500). After washing, membranes were incubated with HRP-conjugated secondary antibodies at room temperature. Protein bands were visualized using an enhanced chemiluminescence detection system, and band intensities were quantified with ImageJ software. Expression levels of target proteins were normalized to β-actin.

### 4.6. Gut Microbiota–Metabolite Axis Analysis

#### 4.6.1. 16S rRNA Gene Sequencing and Microbiota Analysis

Fresh fecal samples were collected under sterile conditions, snap-frozen in liquid nitrogen, and stored at −80 °C until analysis. Total microbial DNA was extracted using a commercially available kit following the manufacturer’s instructions. The V3–V4 region of the bacterial 16S rRNA gene was amplified by PCR using universal primers. After purification and quality assessment, the amplicons were sequenced on the Illumina NovaSeq 6000 platform. Raw sequencing data were subjected to quality filtering, sequence assembly, and operational taxonomic unit (OTU) clustering or amplicon sequence variant (ASV)-based analysis according to established bioinformatics workflows. Subsequent analyses included species annotation, α-diversity and β-diversity evaluation, taxonomic composition analysis, and visualization of microbiota abundance.

#### 4.6.2. Targeted Metabolomics Detection of Short-Chain Fatty Acids (SCFAs)

Fecal samples were thawed on ice, homogenized, and metabolites were extracted according to a standardized protocol. After centrifugation, the supernatants were collected and derivatized prior to analysis. SCFAs were quantified using UPLC-MS/MS under specified chromatographic and mass spectrometric conditions. Concentrations of individual SCFAs were calculated based on corresponding standard curves and normalized for intergroup comparisons. Pearson correlation coefficient analysis was conducted to assess correlations between differential GM and SCFAs, and a correlation heatmap was generated accordingly.

### 4.7. Statistical Analysis

Statistical analyses were performed using SPSS 27.0 and GraphPad Prism 8.0.2. Normally distributed data were analyzed by one-way ANOVA with LSD or Dunnett’s T3 post hoc tests and presented as mean ± SD. A *p*-value < 0.05 was considered statistically significant.

## 5. Conclusions

In conclusion, this study clarifies that the pharmacodynamic material basis of LHHZ against CAG resides in the active components absorbed in gastrointestinal tissues. It was demonstrated that LHHZ exerted significant protective effects in a mouse model of CAG by ameliorating gastric mucosal injury and restoring gastric functional markers. On one hand, LHHZ targets and regulates core molecules, including IL-1β and TNF-α, via its key active components (e.g., berberine), suppresses activation of the IL-17 signaling pathway, and decreases the release of pro-inflammatory factors to mitigate gastric mucosal inflammation. On the other hand, LHHZ can improve intestinal microecological disorders by modulating GM composition (reducing the F/B ratio, restoring the abundance of SCFA-producing bacteria) and normalizing SCFA metabolism, thereby synergistically alleviating gastric mucosal injury. This study provides preclinical evidence supporting further investigation of LHHZ and offers new insights into the mechanistic research of TCM compounds and multi-target approaches against CAG.

## Data Availability

Data will be made available on request.

## Supplementary Materials

The following supporting information can be downloaded at: https://www.mdpi.com/article/10.3390/ph19071043/s1, Figure S1: The results of molecular docking (A) Heatmap (B) Results of molecular docking; Figure S2: Histogram of LDA value distribution. Figure S3: Correlation Heatmap: The right side of the heatmap displays microbial classifications, while the bottom shows metabolites. Dendrograms for hierarchical clustering are plotted on the left and top based on correlation coefficients. The color bar represents the magnitude of the correlation coefficient, where values closer to an absolute value of 1 indicate stronger metabolite correlations. Red denotes positive correlations, while blue indicates negative correlations, with darker shades representing stronger correlations. Asterisks signify significant correlations between metabolites and microorganisms. (* *p* < 0.05, ** *p* < 0.01, *** *p* < 0.001.); Table S1: Chemical component identification of traditional Chinese medicine samples, gastric tissue and intestinal tissue under positive and negative ion modes; Table S2: Specific forward and reverse primer sequences of target genes for quantitative real-time PCR analysis.
