# Supplementary material for: Mechanism of Lian-Huo-Hua-Zhuo Formula in Alleviating Gastric Mucosal Inflammation in a Mouse Model of Chronic Atrophic Gastritis by Inhibiting the IL-17 Signaling Pathway
Source: Pharmaceuticals (Basel). 2026 Jul 5;19(7):1043. doi: 10.3390/ph19071043 (PMC13415115; doi:10.3390/ph19071043)
Supplement: Supplementary file 1 [file pharmaceuticals-19-01043-s001.zip › Supplementary Table S1.pdf]

Table S1-1 Identification results of chemical components of traditional Chinese medicine samples under positive and negative ion mode.

No.	Ion Mode	t_R /min	Adducts	Theoretical m/z	Experimental m/z	Fragments	Mass Error ($\times 10^{-6}$)	Formula	English Name	class
1	POS	4.44	$[M+NH_4]^+$	166.0862	166.0861	120.081, 103.0548	-0.77	C9H8O2	Cinnamic acid	Cinnamic acids and derivatives
2	POS	5.09	$[M+H-H_2O]^+$	181.0495	181.0493	72.0819, 70.0663, 116.0709, 144.9971, 55.0556, 130.9817, 91.055, 53.0401, 86.0609, 95.0498	-1.15	C9H10O5	2,3,4-trihydroxybenzenepropionic acid	Phenylpropanoic acids
3	NEG	5.43	$[M-H]^-$	315.0721	315.0725	108.0196, 123.0432, 153.054, 315.0739, 152.0097, 109.0277, 153.0172	1.03	C13H16O9	3-carboxy-4-hydroxy-phenoxy glucoside	Organooxygen compounds
4	POS	5.95	$[M+H]^+$	153.0546	153.0545	107.0497, 135.0439, 79.0553, 84.9606, 95.0501, 153.054	-1.10	C8H8O3	Isovanillin	Phenols
5	NEG	7.12	$[M-H]^-$	353.0878	353.0880	191.0551, 135.0429, 179.0337, 353.0877, 85.027, 134.0355	0.74	C16H18O9	Heriguard	Organooxygen compounds
6	POS	10.16	$[M+H-H_2O]^+$	177.0546	177.0544	145.0284, 177.0545, 117.0339, 149.0597, 105.9353, 89.0394, 151.0389, 55.9359, 123.9453, 163.0389	-0.92	C10H10O4	Cis-ferulic acid	Cinnamic acids and derivatives
7	POS-NEG	10.34	$[M-H]^-$	353.0878	353.0879	135.0431, 173.0443, 191.0551, 179.0338, 93.0323, 353.0883, 85.027, 111.0431	0.40	C16H18O9	Chlorogenic acid	Organooxygen compounds
8	POS-NEG	11.18	$[M+NH_4]^+$	640.2236	640.2230	163.0388, 85.0295, 71.0504, 135.0447, 145.0281, 107.0495, 325.0903	-0.85	C29H34O15	Isocrenatoside	Cinnamic acids and derivatives
9	NEG	11.4	$[M-H]^-$	457.135	457.135	119.0481, 163.0384, 457.1378, 87.3776,	1.32	C20H26	Regaloside e	-

		6		1	7	385.1294, 68.7065, 70.7197, 86.5189, 89.3698, 85.4646		O12		
10	NEG	11.77	$[M+FA-H]^-$	335.0772	335.0777	135.0434, 179.0341, 161.0226, 335.0777, 93.0324, 133.0277	1.23	C15H14O6	Javanicin	Naphthalenes
11	NEG	12.51	$[M-H]^-$	429.1402	429.1396	173.0444, 175.0385, 134.035, 61.986, 160.0148, 193.0495, 367.105, 191.0551, 429.1408, 93.0322	-1.38	C19H26O11	Regaloside f	Glycerolipids
12	POS	12.84	$[M+NH4]^+$	356.1856	356.1850	206.1172, 356.1859, 58.0666, 191.0934, 190.0858, 356.1449	-1.72	C21H22O4	Licochalcone a	Linear 1,3-diarylpropanoids
13	NEG	13.09	$[M-H]^-$	639.1930	639.1935	161.0225, 133.0275, 639.1931, 179.0337, 135.0433, 151.038, 123.0433, 113.0222, 150.0301	0.71	C29H36O16	Orobanchoside	-
14	POS-NEG	13.11	$[M+H]^+$	239.0914	239.0910	147.0439, 119.0494, 91.0551	-1.76	C12H14O5	Regaloside d deglycosylation	Cinnamic acids and derivatives
15	POS	13.13	$[M]^+$	388.0902	388.0909	177.0545, 145.0283, 117.0339, 287.0446, 89.0395, 311.0897, 85.0294, 329.0435, 56.9661, 149.0593	1.97	C18H16N2O8	Betanidin	Indoles and derivatives
16	POS	13.15	$[M+H]^+$	209.0809	209.0806	145.0284, 177.0545, 117.0339, 149.0596, 89.0394, 91.0552, 134.0364	-1.23	C11H12O4	Ethyl caffeate	Cinnamic acids and derivatives
17	POS	13.35	$[M+NH4]^+$	354.0972	354.0968	206.1173, 354.0961, 311.0905, 294.0752, 336.0873, 352.1168, 353.122, 310.1063, 338.1007, 278.0811	-1.01	C19H12O6	Pachyrhizin	Isoflavonoids

18	POS	14.10	$[M+H]^+$	208.0968	208.0966	208.0966, 151.0752, 91.0551, 119.0494, 171.0287, 177.0537, 165.0909, 145.0284, 117.0339, 95.0499	-1.12	C11H13NO3	Corydaldine	Isoquinolines and derivatives
19	POS	14.25	$[M+Na]^+$	374.1574	374.1591	255.0647, 329.1016, 222.1118, 374.1567, 314.0771, 70.0663, 223.1159, 58.0665, 227.0693, 254.0572	4.66	C18H25NO6	(15e)-12,18-dihydroxysenecionan-11,16-dione	Macrolides and analogues
20	POS	14.41	$[M+H]^+$	192.0655	192.0652	192.0654, 149.0597, 119.0495, 91.0551, 174.0552, 105.0705, 147.0436	-1.99	C10H9NO3	Noroxyhydrastinine	Isoquinolines and derivatives
21	NEG	14.63	$[M-H]^-$	267.0874	267.0875	133.0276, 267.0878, 160.0149, 252.0635, 134.0354, 175.0388, 123.0428, 177.0184, 91.5899, 156.2249	0.30	C13H16O6	1-o-feruloylglycerol	Cinnamic acids and derivatives
22	POS	14.67	$[M]^+$	518.1783	518.1803	307.0832, 322.1065, 518.1828, 279.0879, 91.0582, 320.0909	3.86	C26H30O11	3-(3,4-dimethoxyphenyl)-2,8-dimethoxy-6-[(2r,3r,4r,5r,6r)-3,4,5-trihydroxy-6-(hydroxymethyl)oxan-2-yl]oxy-4h-naphthalen -1-one	Naphthalenes
23	NEG	14.70	$[M-H]^-$	359.1500	359.1504	160.0516, 329.1414, 175.0745, 161.0591, 159.0438, 178.0615, 121.0275, 163.0381, 158.0345, 177.0551	1.21	C20H24O6	Lariciresinol	Furanoid lignans
24	NEG	15.21	$[M-H]^-$	327.1238	327.1240	149.0228, 295.0804, 312.1001, 121.0273, 327.1254, 164.0465, 325.13, 310.1065, 311.1082, 271.6091	0.75	C19H20O5	Columbianadin	Coumarins and derivatives
25	NEG	15.2	$[M-H]^-$	653.208	653.209	161.0229, 135.043, 179.0337, 133.0276,	1.61	C30H38	Campneoside	Cinnamic

		2		7	7	653.2078, 151.0387, 123.0422, 150.0309, 113.0222		O16		acids and derivatives
26	POS	15.34	$[M]^+$	320.0918	320.0909	320.0911, 292.0964, 277.0727, 262.0856, 318.0754, 290.0805	-2.62	C19H14NO4+	Coptisine	-
27	NEG	15.37	$[M-H]^-$	398.1609	398.1610	323.1181, 216.4938, 186.4716, 92.9578, 142.9607, 74.0709, 71.0115, 71.2248	0.24	C22H25NO6	Colchicine	Tropones
28	NEG	15.68	$[M-H]^-$	431.0984	431.0983	78.9567, 311.0571, 283.0616, 431.1002, 91.5825, 141.0701, 429.2, 229.771, 252.3365, 71.0117	-0.12	C21H20O10	Cosmetin	Flavonoids
29	POS	15.70	$[M+H-H2O]^+$	147.0440	147.0438	119.0495, 91.0551, 147.0439, 84.9607	-1.41	C9H8O3	P-coumaric acid	Cinnamic acids and derivatives
30	POS	16.08	$[M]^+$	322.1074	322.1072	307.0832, 322.1066, 163.0384, 279.0879, 321.0944	-0.87	C19H16NO4+	Groenlandicine	-
31	NEG	16.25	$[M-H]^-$	461.0725	461.0731	285.0412, 461.0711, 61.986	1.14	C21H18O12	Scutellarin	Flavonoids
32	POS	16.34	$[M+H-H2O]^+$	163.0389	163.0387	135.044, 163.0387, 89.0394, 117.0339, 145.0283, 107.0497, 79.0552	-1.29	C9H10O5	Danshensu	Phenylpropanoic acids
33	POS-NEG	16.36	$[M-H]^-$	623.1981	623.1985	161.0228, 133.0276, 623.1995, 135.0433, 113.0223, 461.1671, 85.0272	0.58	C29H36O15	Acteoside	Cinnamic acids and derivatives
34	POS	16.36	$[M]^+$	338.1387	338.1378	338.1372, 322.1046, 294.1114, 323.113, 308.091, 280.0953, 279.0874, 306.1122, 307.0834, 321.0944	-2.68	C20H20NO4+	Jatrorrhizine	-
35	POS-NEG	16.40	$[M-H]^-$	338.1398	338.1399	293.0698, 308.0929, 323.118, 338.1399, 265.0743, 264.0668, 292.0624	0.52	C20H21NO4	(r)-canadine	Protoberberine alkaloids and

										derivatives
36	NEG	16.66	$[M-H]^-$	447.0933	447.0936	285.0409, 284.0331, 447.0917, 61.9858, 133.0259	0.78	C21H20O11	Scutellarein-5-galactoside	Flavonoids
37	POS	16.80	$[M+H-H2O]^+$	334.1074	334.1073	320.091, 292.096, 321.0988, 306.0755, 304.0955, 278.0804	-0.18	C20H17NO5	Berlambine	Isoquinolines and derivatives
38	POS	16.82	$[M+H]^+$	322.1074	322.1071	307.0831, 322.1064, 163.0387, 135.0437, 292.0964, 321.0954, 145.0282, 320.0908	-0.99	C19H15NO4	Berberrubine	Isoquinolines and derivatives
39	POS	16.88	$[M]^+$	336.1231	336.1222	336.1221, 292.0953, 320.0912, 321.0973, 306.0756, 278.0794, 304.0959, 322.1065, 294.1116, 323.1144	-2.62	C20H18NO4+	Berberine	-
40	POS	17.68	$[M]^+$	352.1544	352.1537	352.1534, 336.1222, 308.1271, 337.1284, 294.1108, 322.105, 320.1263, 292.0929, 320.0922, 321.0952	-2.03	C21H22NO4+	Palmatine	-
41	POS	17.70	$[M+H]^+$	216.1747	216.1745	216.1744, 160.1119, 174.1275, 161.1187, 146.0963, 145.0881, 144.0803, 132.0808	-0.77	C15H21N	Epiguaipyridine	Pyridines and derivatives
42	NEG	17.96	[M+FA-H]-	625.2138	625.2141	417.1556, 161.023, 166.0258, 181.049, 59.0114, 457.568, 144.4905, 394.3507, 99.7341, 465.255	0.47	C28H36O13	Acanthoside b	Lignan glycosides
43	NEG	18.03	$[M-H]^-$	269.0455	269.0452	269.0468, 117.0328, 63.0216	-1.29	C15H10O5	Apigenin	Flavonoids
44	POS	18.14	$[M+H-H2O]^+$	177.0546	177.0545	117.0339, 145.0283, 89.0395, 177.0544, 149.0597, 134.0362	-0.47	C10H10O4	Fer	Cinnamic acids and derivatives
45	POS	18.36	$[M+H-H2O]^+$	311.1278	311.1276	137.0596, 219.0802, 191.0849, 251.1062, 311.1282, 190.0776, 279.1002, 220.0864,	-0.57	C19H20O5	Columbianadin	Coumarins and

						218.0723, 187.075				derivatives
46	POS	18.49	$[M+H]^+$	357.1333	357.1327	137.0599, 307.0943, 247.0749, 219.0801, 177.0549, 55.0193, 86.0974, 145.0284, 122.0365, 191.0851	-1.70	C20H20O6	3-(3,4-dimethoxyphenyl)-6-hydroxy-2,8-dimethoxy-4h-naphthalen-1-one	Naphthalenes
47	POS	18.61	$[M+H-H2O]^+$	368.1492	368.1489	368.1484, 338.1014, 310.1066, 352.1174, 353.1244, 324.1227, 336.1218, 308.0915, 337.0895	-1.04	C21H23NO6	3-demethylcolchicine	Tropones
48	NEG	18.84	$[M-H]^-$	693.2036	693.2046	160.015, 175.0387, 693.2055, 132.0197, 134.0354, 193.0494	1.39	C32H38O17	3,6'-o-diferuloylsucrose	-
49	POS	20.18	$[M+H]^+$	267.1591	267.1586	70.0664, 55.0557, 101.0606, 81.071, 119.0861, 95.0865, 121.1016, 93.0709, 107.0863, 105.0704	-1.86	C15H22O4	Magnograndiolide	Prenol lipids
50	POS-NEG	20.26	[M+NH4]+	460.1813	460.1807	308.0913, 336.0865, 237.0291, 320.09, 481.1069, 105.0705, 98.9757, 85.0294, 323.1129, 387.0956	-1.31	C20H26O11	Regaloside b	-
51	POS	20.44	$[M+H-H2O]^+$	507.1861	507.1856	145.0285, 177.0547, 117.0339	-0.95	C25H32O12	6-o-e-feruloylajugol	-
52	NEG	20.83	[M+FA-H]-	505.1352	505.1356	327.0513, 313.0361, 505.1335, 256.0377, 299.0564, 284.0327, 490.1146, 165.0181, 343.084, 285.0409	0.85	C23H24O10	Irisolidone-7-o-α-l-rhamnopyranoside	Isoflavonoids
53	NEG	21.26	[M+FA-H]-	301.0718	301.0717	301.0713, 151.0014, 134.0352, 65.0007, 107.0114, 164.0101, 78.9565, 149.0585, 108.0191, 136.0142	-0.12	C15H12O4	Emodin anthrone	Anthracenes
54	NEG	22.18	$[M-H]^-$	399.1297	399.1296	137.0595, 99.0065, 399.1293, 163.0385, 125.0589, 71.0113, 91.5847, 194.5504,	-0.15	C18H24O10	Regaloside a	Glycerolipids

						95.048, 206.0756				
55	NEG	25.43	$[M-H]^-$	459.0933	459.0938	121.0276, 128.0335, 401.0863, 313.073, 356.9972, 371.0009, 304.7947, 154.8143, 174.9603, 240.7616	1.07	C22H20O11	Wogonoside	Flavonoids
56	POS-NEG	25.62	$[M+H]^+$	237.1849	237.1844	219.1739, 81.0708, 107.086, 137.0957, 159.1163, 93.0707, 123.0807, 95.0864, 55.0557, 201.1638	-2.30	C15H24O2	10alpha-hydroperoxy-guaia-1,11-diene	Prenol lipids
57	POS	25.66	$[M+H-H2O]^+$	415.3207	415.3199	81.0709, 95.0863, 352.081, 397.3101, 93.0709, 57.0714, 105.0704, 67.0555, 145.1008, 131.0852	-1.90	C27H44O4	26-o-beta-d-glucopyranosyl-3beta,26-dihydroxy-choleslen-16,22-dioxo-3-o-alpha-l-rhamnopyranosyl-(1-2)-beta-d-glucopyranoside deglycosylation	-
58	NEG	26.03	$[M-H]^-$	265.1445	265.1443	265.1458, 221.1549, 96.9578, 216.9892, 131.0118, 178.9913, 264.9903, 244.9837, 114.0667, 264.1581	-0.65	C15H22O4	Magnograndiolide	Prenol lipids
59	NEG	26.53	$[M-H]^-$	329.0667	329.0668	299.0202, 171.1012, 314.0443, 271.0255, 211.1326, 329.0671, 139.1107, 229.1442, 99.0792, 127.1106	0.43	C17H14O7	Ombuin	Flavonoids
60	POS	27.10	$[M+H-H2O]^+$	368.1492	368.1485	338.1013, 368.1494, 310.1065, 353.126, 95.0862, 81.071, 67.0554	-1.97	C21H23NO6	3-desmethylcolchicine	Tropones
61	NEG	27.61	$[M-H]^-$	283.0612	283.0612	268.0382, 283.0625, 78.9567, 114.09	0.05	C16H12O5	Genkwanin	Flavonoids

62	POS	27.65	$[M+H]^+$	165.0910	165.0908	84.9607, 119.0193, 137.0293, 86.9937, 105.0039, 68.9834, 85.0294, 104.9639, 117.9458, 118.9677	-1.32	$C_{10}H_{12}O_2$	P-hydroxybenzyl acetone	Phenols
63	NEG	28.92	[M+FA-H]-	475.3065	475.3063	475.3083, 78.9566, 96.967	-0.49	$C_{27}H_{42}O_4$	26-o-β-d-glucopyranosyl -nuatigenin -3-o-α-l-rhamnopyranosyl(1→2) -β-d-glucopyranoside deglycosylation	-
64	POS	28.94	$[M+H]^+$	237.1849	237.1845	107.0861, 95.0863, 121.1014, 119.0859, 237.184, 81.0708, 105.0704, 179.1427, 93.0707, 109.1018	-1.89	$C_{15}H_{24}O_2$	15α-hydroperoxy-guaia-1(10),11-diene	Prenol lipids
65	NEG	29.36	[M+FA-H]-	263.1653	263.1653	263.1656, 131.0116, 130.0151, 205.1591, 219.175, 235.1702, 160.0877	0.09	$C_{15}H_{22}O$	1,2,3,4,5,6 -hexahydro -1,1,5,5-tetramethyl -7h-2,4a-methano naphthalen -7-one	Prenol lipids
66	POS	30.35	$[M+H]^+$	127.0390	127.0390	127.0391, 99.0446, 82.0662, 109.0289, 71.0503, 81.0344, 59.0504	0.04	$C_6H_6O_3$	5-hydroxymethylfurfural	Organooxygen compounds
67	POS-NEG	30.35	$[M+H]^+$	225.1122	225.1115	81.0709, 139.0389, 85.0295, 207.1013, 225.1116, 127.0391, 71.0867, 99.081, 95.0866, 79.0555	-2.92	$C_{12}H_{16}O_4$	Pogostone	Organooxygen compounds
68	NEG	30.37	[M+FA-H]-	515.1923	515.1922	207.1021, 123.0431, 139.0746, 163.1116, 219.1021, 471.2037, 78.9566, 515.1876, 223.0971, 135.0433	-0.11	$C_{26}H_{30}O_8$	Limonin	Prenol lipids

69	POS	30.39	$[M+H-H2O]^+$	149.0961	149.0959	84.9606, 149.0959, 107.086, 149.0236, 105.0705, 102.9707, 93.0707, 79.0553, 121.0287, 91.0552	-1.39	C10H14O2	Perilla ketone	Organooxygen compounds
71	POS-NEG	30.54	$[M+H]^+$	345.0969	345.0963	345.0961, 329.065, 330.0721, 301.0699, 240.2318, 315.0489, 57.0713, 287.0536, 167.0336	-1.67	C18H16O7	Pachypodol	Flavonoids
70	POS	30.94	$[M+H]^+$	359.1126	359.1117	383.093, 369.0776, 81.0707, 91.577, 382.0977, 93.0706, 118.4829, 67.0554, 55.0556, 106.5827	-2.35	C19H18O7	Retusin	Flavonoids
72	NEG	31.49	$[M-H]^-$	269.0455	269.0457	269.046, 62.9617, 225.0551, 79.9549, 225.1868	0.69	C15H10O5	Emodin	Anthracenes
73	POS-NEG	31.71	$[M+H]^+$	299.0914	299.0909	299.0908, 256.0728, 284.0675, 67.0555	-1.73	C17H14O5	7-hydroxy-5,8-dimethoxyflavone	Flavonoids
74	POS	31.82	$[M+H-H2O]^+$	313.1070	313.1064	298.0828, 313.1065, 297.0752, 95.0863, 255.0639, 67.0555, 270.088, 81.071, 93.0706, 55.0557	-2.02	C18H18O6	5-hydroxy-7,3‘,4'-trimethoxyflavanone	Flavonoids
75	POS-NEG	32.37	$[M+H]^+$	315.0863	315.0857	315.0848, 272.067, 300.0605, 167.0337, 244.0736, 121.0656, 57.0713, 135.0439	-2.12	C17H14O6	3,5-dihydroxy-4',7-dimethoxyflavone	Flavonoids
76	POS	33.03	[M+Na]+	487.3394	487.3413	119.0859, 95.0863, 107.0861, 105.0706, 133.1013, 145.101, 487.3465, 121.1015, 93.0708, 147.1164	4.03	C28H48O5	Trihydroxybufosterocholanic acid	Steroids and steroid derivatives
77	POS	33.05	$[M+H-H2O]^+$	149.0233	149.0232	84.9607, 149.0233, 121.0287, 65.0399, 102.9708, 121.0398, 93.0343	-0.92	C8H6O4	Phthalate	Benzene and substituted derivatives
78	POS	34.42	$[M+H-H2O]^+$	161.1324	161.1322	116.9723, 105.0705, 117.9801, 119.0858, 161.1324, 91.0551, 114.9564, 132.9668, 79.0557, 57.9362	-1.28	C12H20O2	Alpha-terpinyl acetate	Prenol lipids

79	POS	34.42	$[M+H-H2O]^+$	147.1168	147.1166	84.9607, 147.1166, 105.0705, 119.0859, 102.9708, 91.0551	-1.56	C11H16O	Tricyclo[4.3.1.12,5]undec-3-en-10-ol	Organooxygen compounds
80	POS	34.44	$[M+H]^+$	221.1900	221.1896	95.0863, 81.0709, 107.086, 221.1895, 109.1018, 203.1794, 119.0857, 105.0704, 147.1165, 69.0711	-1.84	C15H24O	(+)-guaia-10(15),11-dien-1-alpha-ol	Prenol lipids
81	NEG	36.57	[M+FA-H]-	529.3018	529.3019	279.2333, 529.3036, 85.027, 113.0221, 325.1844, 59.0114, 249.0619, 78.9566, 255.2343, 75.0063	0.10	C26H44O8	Darutoside	Prenol lipids
83	NEG	38.15	[M+FA-H]-	459.3116	459.3118	109.0637, 415.3226, 459.3126, 343.3007, 61.986, 325.1842, 91.5839, 78.9566, 397.3086, 69.0323	0.36	C27H42O3	Ophipogonin d deglycosylation	Prenol lipids
82	POS	40.49	$[M+H-H2O]^+$	395.3672	395.3665	81.0709, 95.0863, 145.1012, 395.3662, 69.0712, 67.0555, 105.0705, 159.1167, 133.1011, 57.0713	-1.85	C29H48O	Stigmasta-5,22-dien-3-ol	Steroids and steroid derivatives
84	POS	41.28	$[M+H]^+$	303.2319	303.2310	81.0708, 93.0706, 95.0863, 67.0555, 121.1011, 79.0553, 302.3046, 91.5796, 57.0713, 69.0711	-2.91	C20H30O2	Isopimaric acid	Prenol lipids
85	POS	42.63	$[M+H-H2O]^+$	397.3829	397.3819	81.0709, 95.0862, 397.3822, 147.1167, 109.1016, 67.0555, 161.1323, 105.0704,	-2.32	C29H50O	Delta(7)-stigmastenol	Steroids and steroid derivatives

Table S1-2 Identification of gastric histochemical composition in positive and negative ion mode.

**No.**	**Ion Mode**	**t_R /min**	**Adducts**	**Theoretical m/z**	**Experimental m/z**	**Fragments**	**Mass Error ($\times 10^{-6}$)**	**Formula**	**English Name**	**Source**
1	POS	4.63	$[M+H]^+$	211.0600	211.0601		-0.56	C10H10O4	Cis-ferulic acid	*Coptis chinensis*

2	NEG	6.46	$[M+FA-H]^-$	313.0567	313.0565	137.0227, 287.9296, 127.8941, 90.36, 187.635, 149.4456, 192.2372, 115.9988, 70.0265	0.66	C12H14O5	Regaloside d deglycosylation	*Lilium lancifolium*
3	POS	6.73	$[M+H]^+$	187.0601	187.0601	146.0599, 118.0655, 144.0807, 170.0596, 143.0729	-0.20	C9H10O5	2,3,4-trihydroxybenzenepropionic acid	*Coptis chinensis*
4	POS	7.21	$[M+H]^+$	240.0864	240.0866	87.0448, 121.0846, 81.071, 154.0507, 70.066, 151.021, 75.2953, 120.0816, 139.9806, 180.1989	-1.17	C11H13NO3	Corydaldine	*Coptis chinensis*
5	NEG	9.02	$[M-H]^-$	299.0773	299.0772	137.0224, 93.0322, 280.0866, 282.0847, 297.1127, 112.086, 257.0882, 138.0544, 141.0094, 158.4265	0.17	C13H16O6	1-o-feruloylglycerol	*Lilium lancifolium*
6	NEG	9.45	$[M-H]^-$	343.1034	343.1035	167.0699, 343.1048, 59.0114, 85.0273, 113.0224, 152.0456, 71.0116, 92.9174, 137.0588, 99.0065	-0.26	C16H18O9	Heriguard	*Coptis chinensis*
7	POS	9.99	$[M-e]^-$	328.1539	328.1544	70.0663, 58.0666, 98.9848, 84.0817, 86.0973, 120.0811, 110.0717, 72.0819, 328.155, 84.0454	-1.50	C20H20NO4+	Jatrorrhizine	*Pogostemon cablin* *Coptis chinensis*
8	NEG	10.24	$[M-H]^-$	473.1297	473.1301	161.0229, 135.043, 179.0329, 353.0877, 251.0566, 389.086, 250.9874, 311.0772, 134.0356, 249.9392	-0.70	C20H26O11	Regaloside b	*Lilium lancifolium*
9	NEG	10.60	$[M+FA-H]^-$	399.0933	399.0933	223.0604, 193.0131, 149.0228, 95.2205, 399.0953, 58.4233, 339.786, 179.7966, 150.1246, 64.8422	0.11	C16H18O9	Chlorogenic acid	*Coptis chinensis*

10	NEG	11.11	[M-H]-	430.1509	430.1507		0.39	C22H25NO6	Colchicine	*Lilium lancifolium*
11	NEG	11.49	[M-H]-	457.1356	457.1352		1.06	C20H26O12	Regaloside e	*Lilium lancifolium*
12	NEG	11.68	[M+FA-H]-	317.0667	317.0667		-0.09	C15H10O5	Apigenin	*Pogostemon cablin*
13	NEG	11.80	[M-H]-	643.2254	643.2244	357.1347, 327.0886, 181.0495, 166.0254, 312.0651, 405.1563, 342.1111, 329.0429, 608.2508, 193.0512	1.603219745	C30H38O16	Campneoside	*Pogostemon cablin*
14	POS	11.91	[M+H]+	208.0603	208.0604		-0.43	C10H9NO3	Noroxyhydrastinine	*Coptis chinensis*
15	POS	12.11	[M-e]-	338.1015	338.1023		-2.52	C19H14NO4+	Coptisine	*Pogostemon cablin*
16	NEG	12.29	[M-H]-	313.0568	313.0565	121.0272, 197.0443, 153.055, 71.0115, 228.6478, 65.1978, 135.1761, 182.0385, 141.6869, 103.6561	0.86	C13H16O9	3-carboxy-4-hydroxy-phenoxy glucoside	*Coptis chinensis*
17	NEG	12.44	[M-H]-	449.1086	449.1089	269.0461, 125.0222, 117.0324, 449.1091, 99.9231, 376.6499, 83.0118, 232.8297, 124.6797, 125.5064	-0.65	C21H20O10	Cosmetin	*Pogostemon cablin*
18	POS	12.47	[M-e]-	388.0905	388.0902		0.96	C18H16N2O8	Betanidin	*Lilium lancifolium*
19	NEG	12.51	[M-H]-	429.1397	429.1402	175.0391, 134.0351, 160.0148, 193.0496, 429.1397, 173.0443, 425.046, 69.1352, 224.5564, 61.986	-1.19	C19H26O11	Regaloside f	*Lilium lancifolium*
20	NEG	12.6	[M+FA-	335.077	335.077	135.0431, 179.0333, 161.0226, 91.5894,	0.51	C15H14	Javanicin	*Coptis*

		6	H]$^-$	4	2	93.0323, 244.3693, 247.4626, 278.0766, 306.9011, 195.1232		O6		*chinensis*
21	POS	12.67	[M+H]$^+$	521.2012	521.2017	131.0492, 103.0548, 70.0663, 84.0818, 86.0973, 84.0454, 163.0751, 110.0715, 91.0584, 72.0821	-1.03	C26H30O11	3-(3,4-dimethoxy phenyl) -2,8-dimethoxy -6-[(2r,3r,4r,5r,6r) -3,4,5-trihydroxy -6-(hydroxymethyl)oxan -2-yl]oxy-4h-naphthalen -1-one	*Pogostemon cablin*
22	POS	13.01	[M-e]$^-$	498.1389	498.1395	307.0835, 70.0664, 322.1071, 84.0817, 72.082, 110.0719, 120.0779, 86.0975, 84.0451	-1.22	C19H16NO4+	Groenlandicine	*Coptis chinensis*
23	NEG	13.21	[M-H]$^-$	621.1830	621.1825	621.1838, 269.082, 91.5884, 267.0658, 572.9731, 511.4493, 209.4342, 478.4173, 273.4265, 136.7046	0.87	C29H34O15	Isocrenatoside	*Pogostemon cablin*
24	POS	13.55	[M+H]$^+$	310.1066	310.1074	295.0836, 310.1067, 308.0916, 267.088	-2.50	C19H15NO4	Berberrubine	*Coptis chinensis*
25	POS	13.77	[M+H]$^+$	388.1743	388.1755		-3.04	C21H23NO6	3-demethylcolchicine	*Lilium lancifolium*
26	POS	13.91	[M+H]$^+$	248.1643	248.1645	202.159, 248.165, 84.9605, 146.0964, 160.1118, 105.0705, 93.0707, 230.1551, 91.0552, 161.0965	-0.71	C15H21N	Epiguaipyridine	*Pogostemon cablin*
27	POS	14.58	[M+H]$^+$	340.1535	340.1543	70.0663, 324.1225, 325.1283, 340.1546, 145.028, 177.0547, 310.1433, 137.0597, 86.0973, 58.0664	-2.36	C20H21NO4	(r)-canadine	*Coptis chinensis*

28	POS	14.67	$[M-e]^{-}$	354.1326	354.1336	337.0897, 309.0945, 353.1206, 354.1253, 189.0782, 321.0948, 295.0779, 188.0704, 323.074, 338.1377	-2.91	C20H18NO4+	Berberine	*Coptis chinensis*
29	NEG	14.69	$[M-H]^{-}$	359.1503	359.1500		0.89	C20H24O6	Lariciresinol	*Coptis chinensis*
30	NEG	15.14	$[M-H]^{-}$	595.2040	595.2032	389.1621, 85.0269, 595.2026, 175.0384, 113.022, 71.0115, 468.2851, 190.1607, 59.0114, 205.2039	1.21	C28H36O13	Acanthoside b	*Pogostemon cablin*
31	POS	15.16	$[M+H]^{+}$	373.1270	373.1282		-3.10	C20H20O6	3-(3,4-dimethoxyphenyl)-6-hydroxy-2,8-dimethoxy-4h-naphthalen-1-one	*Pogostemon cablin*
32	POS	15.30	$[M+H]^{+}$	327.1581	327.1591	325.1255, 310.1008, 309.0942, 295.0788, 281.0993, 98.985, 267.0837, 137.0598, 293.0988, 280.0956	-3.10	C21H22O4	Licochalcone a	*Pogostemon cablin*
33	POS	15.83	$[M+H]^{+}$	340.1177	340.1180	145.0282, 177.0544, 340.1177, 117.0338, 137.0598, 310.0703, 86.0974, 70.0665, 290.0795, 308.0916	-0.78	C20H17NO5	Berlambine	*Coptis chinensis*
34	POS	15.95	$[M+H]^{+}$	331.1527	331.1540	86.0974, 70.0663, 98.9847, 137.0595, 151.0749, 57.0713, 224.2958, 249.2567, 91.0549, 218.6808	-3.84	C19H20O5	Columbianadin	*Pogostemon cablin* *Coptis chinensis*
35	POS	16.78	$[M-e]^{-}$	352.1527	352.1544	336.1229, 352.1542, 322.1065, 320.1278, 352.1453, 86.0975, 307.0847, 321.0976, 120.0814, 70.0659	-4.77	C21H22NO4+	Palmatine	*Pogostemon cablin*
36	NEG	16.9	$[M-H]^{-}$	595.204	595.203	59.0114, 369.1324, 135.0434, 103.8817,	1.43	C29H36	Orobanchoside	*Pogostemon*

		6		1	2	179.0338, 463.3586, 154.4666, 581.0075, 129.2573, 86.3696		O_{16}		*cablin*
37	NEG	19.47	$[M-H]^-$	579.2088	579.2083	466.4382, 285.2582, 469.4026, 135.8494, 61.6657, 70.4419	0.89	$C_{29}H_{36}O_{15}$	Acteoside/verbascoside/kusaginin	*Pogostemon cablin*
38	NEG	19.78	$[M-H]^-$	267.1602	267.1602	267.1596, 57.0324, 267.1326, 226.9941, 178.9916, 79.9551, 247.839, 125.0952, 121.3698, 201.0706	0.24	$C_{15}H_{24}O_2$	10alpha-hydroperoxy -guaia-1,11-diene	*Pogostemon cablin*
39	POS	20.21	$[M+H]^+$	382.1276	382.1285	352.0815, 177.0543, 145.0281, 382.1277, 367.1036, 81.071, 338.1016, 324.0863, 117.0333, 95.0865	-2.44	$C_{21}H_{23}NO_6$	3-desmethylcolchicine	*Lilium lancifolium*
40	NEG	20.88	$[M-H]^-$	707.2203	707.2193	207.1024, 135.0433, 161.0231, 179.0336, 123.0429, 267.2636, 133.0269, 686.0807, 391.972, 591.6018	1.43	$C_{32}H_{38}O_{17}$	3,6'-o-diferuloylsucrose	*Lilium lancifolium*
41	NEG	21.28	$[M+FA-H]^-$	505.1357	505.1352	329.1037, 165.0181, 71.0116, 65.0009, 59.0114, 97.0269, 447.7074, 210.5989, 344.7131, 178.4899	1.05	$C_{23}H_{24}O_{10}$	Irisolidone -7-o-α-l-rhamnopyranoside	*Pogostemon cablin*
42	NEG	21.83	$[M-H]^-$	477.1040	477.1038	299.0198, 165.0179, 271.0246, 134.0356, 314.0432, 315.0884, 315.0503, 477.1042, 149.0592, 244.689	0.26	$C_{22}H_{20}O_{11}$	Wogonoside	*Lilium lancifolium*
43	NEG	23.17	$[M-H]^-$	331.0825	331.0823	178.9978, 166.0263, 193.0125, 151.0016, 138.0303, 175.2528, 165.0181, 74.7958, 191.6293, 91.1336	0.39	$C_{17}H_{14}O_7$	Ombuin	*Pogostemon cablin*
44	POS	23.20	$[M+H]^+$	521.1643	521.1654	359.1116, 301.069, 344.089, 329.0666, 70.0663, 91.5838, 90.7518, 213.1456, 330.6038, 162.6612	-1.95	$C_{25}H_{32}O_{12}$	6-o-e-feruloylajugol	*Coptis chinensis*
45	NEG	23.44	$[M-H]^-$	461.1092	461.1089	283.0254, 255.0303, 298.0499, 461.1101, 299.0566, 227.0338, 213.7502, 349.9243, 101.4048	0.504219243	$C_{21}H_{20}O_{11}$	Scutellarein-5-galactoside	*Lilium lancifolium*

46	NEG	24.53	[M-H]$^{-}$	401.1453	401.1453	139.0745, 99.0065, 401.1458, 139.0847, 261.061	-0.08	C18H24O10	Regaloside a	*Lilium lancifolium*
47	POS	25.23	[M+H]$^{+}$	347.1116	347.1125	151.0755, 167.0338, 163.0386, 135.0434, 209.0443, 273.1115, 301.1052, 122.447, 270.0898, 287.4276	-2.65	C18H18O6	5-hydroxy-7,3‘,4'-trimethoxyflavanone	*Pogostemon cablin*
48	POS	28.03	[M+H]$^{+}$	361.1272	361.1282	167.0337, 163.0385, 91.5773, 70.0663, 57.0713	-2.82	C19H18O7	Retusin	*Pogostemon cablin*
49	NEG	28.35	[M+FA-H]$^{-}$	329.0670	329.0667	171.1015, 329.0686, 299.0217, 271.0244, 201.1127, 314.0441, 327.2183, 199.1331, 165.0178, 211.1334	0.94	C16H12O5	Genkwanin	*Pogostemon cablin*
50	NEG	28.69	[M-H]$^{-}$	247.1337	247.1340	61.9859, 203.1431, 247.1351, 115.0187, 247.0011, 184.0011, 176.9933, 116.9261, 140.9945, 139.9117	-1.29	C15H22O4	Magnograndiolide	*Coptis chinensis*
51	NEG	30.39	[M+FA-H]$^{-}$	515.1923	515.1923		-0.01	C26H30O8	Limonin	*Coptis chinensis*
52	POS	31.39	[M+H]$^{+}$	345.0961	345.0969	345.0952, 329.065, 240.2316, 57.0712, 330.0715, 71.0867, 287.0537, 301.0712, 85.102, 109.1015	-2.34	C18H16O7	Pachypodol	*Pogostemon cablin*
53	POS	31.75	[M+H]$^{+}$	299.0906	299.0914	299.0906, 256.0724, 284.0675, 81.0709, 67.0554, 69.071, 95.0862, 55.0557, 57.0712, 93.0706	-2.60	C17H14O5	7-hydroxy-5,8-dimethoxyflavone	*Lilium lancifolium*
54	POS	32.40	[M+H]$^{+}$	315.0853	315.0863	315.0854, 81.0709, 300.0608, 272.0673, 67.0555, 95.0863, 69.071, 114.0725, 57.0713, 93.0707	-3.13	C17H14O6	3,5-dihydroxy-4',7-dimethoxyflavone	*Pogostemon cablin*
55	NEG	33.45	[M+FA-H]$^{-}$	489.3225	489.3222		0.62	C27H42O4	26-o-β-d-glucopyranosyl-nuatigenin-3-o-α-l-rhamnop	*Lilium lancifolium*

									yranosyl(1→2)-β-d-glucopyranoside deglycosylation	

Table S1-3 Identification of intestinal histochemical components in positive and negative ion mode.

**No.**	**Ion Mode**	**t_R /min**	**Adducts**	**Theoretical m/z**	**Experimental m/z**	**Fragments**	**Mass Error ($\times 10^{-6}$)**	**Formula**	**English Name**	**Source**
1	NEG	6.46	[M+FA-H]$^-$	313.0567	313.0565	137.0227, 287.9296, 127.8941, 90.36, 187.635, 149.4456, 192.2372, 115.9988, 70.0265	0.663458497	Regaloside d deglycosylation	C12H14O5	*Lilium lancifolium*
2	POS	7.28	[M+H]$^+$	368.0975	368.0976		-0.39	Noroxyhydrastinine	C10H9NO3	*Coptis chinensis*
3	NEG	9.02	[M-H]$^-$	299.0773	299.0772	137.0224, 93.0322, 280.0866, 282.0847, 297.1127, 112.086, 257.0882, 138.0544, 141.0094, 158.4265	0.174202489	1-o-feruloylglycerol	C13H16O6	*Lilium lancifolium*
4	NEG	9.29	[M-H]$^-$	355.1037	355.1035	135.0432, 179.0336, 113.0221, 85.027, 146.0352, 59.0113, 71.0112, 355.0692, 179.0697, 164.0462	0.60	Heriguard	C16H18O9	*Coptis chinensis*
5	NEG	9.99	[M-e]$^-$	328.1539	328.1544	70.0663, 58.0666, 98.9848, 84.0817, 86.0973, 120.0811, 110.0717, 72.0819, 328.155, 84.0454	-1.50	Jatrorrhizine	C20H20NO4+	*Pogostemon cablin Coptis chinensis*
6	NEG	10.6	[M+FA-	399.09	399.093	223.0604, 193.0131, 149.0228, 95.2205,	0.11	Chloroge	C16H18O9	*Coptis*

		0	$H]^-$	33	3	399.0953, 58.4233, 339.786, 179.7966, 150.1246, 64.8422		nic acid		*chinensis*
7	NEG	10.74	$[M-H]^-$	388.1773	388.1766		1.93	Colchicine	C22H25NO6	*Lilium lancifolium*
8	NEG	11.49	$[M-H]^-$	457.1356	457.1352		1.06	Regaloside e	C20H26O12	*Lilium lancifolium*
9	NEG	11.68	$[M+FA-H]^-$	317.0667	317.0667		-0.09	Apigenin	C15H10O5	*Pogostemon cablin*
10	POS	12.08	$[M+H]^+$	222.0758	222.0761		-1.41	Corydaldine	C11H13NO3	*Coptis chinensis*
11	NEG	12.11	$[M-e]^-$	338.1015	338.1023		-2.52	Coptisine	C19H14NO4+	*Pogostemon cablin*
12	NEG	12.29	$[M-H]^-$	313.0568	313.0565	121.0272, 197.0443, 153.055, 71.0115, 228.6478, 65.1978, 135.1761, 182.0385, 141.6869, 103.6561	0.86	3-carboxy-4-hydroxy-phenoxy glucoside	C13H16O9	*Coptis chinensis*
13	NEG	12.44	$[M-H]^-$	449.1086	449.1089	269.0461, 125.0222, 117.0324, 449.1091, 99.9231, 376.6499, 83.0118, 232.8297, 124.6797, 125.5064	-0.65	Cosmetin	C21H20O10	*Pogostemon cablin*
14	NEG	12.51	$[M-H]^-$	429.1397	429.1402	175.0391, 134.0351, 160.0148, 193.0496, 429.1397, 173.0443, 425.046, 69.1352, 224.5564, 61.986	-1.19	Regaloside f	C19H26O11	*Lilium lancifolium*
15	NEG	12.66	$[M+FA-H]^-$	335.0774	335.0772	135.0431, 179.0333, 161.0226, 91.5894, 93.0323, 244.3693, 247.4626, 278.0766,	0.51	Javanicin	C15H14O6	*Coptis chinensis*

						306.9011, 195.1232				
16	NEG	13.01	[M-e]$^{-}$	498.1389	498.1395	307.0835, 70.0664, 322.1071, 84.0817, 72.082, 110.0719, 120.0779, 86.0975, 84.0451	-1.22	Groenlandicine	C19H16NO4+	*Coptis chinensis*
17	NEG	13.21	[M-H]$^{-}$	621.1830	621.1825	621.1838, 269.082, 91.5884, 267.0658, 572.9731, 511.4493, 209.4342, 478.4173, 273.4265, 136.7046	0.87	Isocrenatoside	C29H34O15	*Pogostemon cablin*
18	POS	13.91	[M+H]$^{+}$	248.1643	248.1645	202.159, 248.165, 84.9605, 146.0964, 160.1118, 105.0705, 93.0707, 230.1551, 91.0552, 161.0965	-0.71	Epiguaipyridine	C15H21N	*Pogostemon cablin*
19	POS	14.58	[M+H]$^{+}$	340.1535	340.1543	70.0663, 324.1225, 325.1283, 340.1546, 145.028, 177.0547, 310.1433, 137.0597, 86.0973, 58.0664	-2.36	(r)-canadine	C20H21NO4	*Coptis chinensis*
20	NEG	14.67	[M-e]$^{-}$	354.1326	354.1336	337.0897, 309.0945, 353.1206, 354.1253, 189.0782, 321.0948, 295.0779, 188.0704, 323.074, 338.1377	-2.91	Berberine	C20H18NO4+	*Coptis chinensis*
21	NEG	14.69	[M-H]$^{-}$	359.1503	359.1500		0.888765107	Lariciresinol	C20H24O6	*Coptis chinensis*
22	NEG	15.14	[M-H]$^{-}$	595.2040	595.2032	389.1621, 85.0269, 595.2026, 175.0384, 113.022, 71.0115, 468.2851, 190.1607, 59.0114, 205.2039	1.205470603	Acanthoside b	C28H36O13	*Pogostemon cablin*
23	POS	15.16	[M+H]$^{+}$	373.1270	373.1282		-3.10	3-(3,4-dimethoxyphenyl)-6-hydroxy-2,8-dimethoxy-4h-napht	C20H20O6	*Pogostemon cablin*

								halen -1-one		
24	POS	15.30	[M+H]+	327.1581	327.1591	325.1255, 310.1008, 309.0942, 295.0788, 281.0993, 98.985, 267.0837, 137.0598, 293.0988, 280.0956	-3.10	Licochalcone a	C21H22O4	*Pogostemon cablin*
25	POS	15.95	[M+H]+	331.1527	331.1540	86.0974, 70.0663, 98.9847, 137.0595, 151.0749, 57.0713, 224.2958, 249.2567, 91.0549, 218.6808	-3.84	Columbianadin	C19H20O5	*Pogostemon cablin Coptis chinensis*
26	NEG	16.35	[M+FA-H]-	755.2049	755.2040	284.0328, 255.0303, 227.0334, 755.2045, 264.396, 659.6107, 198.4415, 681.4514, 172.3391, 224.225	1.13	3,6'-o-diferuloylsucrose	C32H38O17	*Lilium lancifolium*
27	NEG	16.78	[M-e]-	352.1527	352.1544	336.1229, 352.1542, 322.1065, 320.1278, 352.1453, 86.0975, 307.0847, 321.0976, 120.0814, 70.0659	-4.77	Palmatine	C21H22NO4+	*Pogostemon cablin*
28	NEG	16.93	[M-H]-	439.1249	439.1246	247.0978, 305.1025, 395.1366, 219.1024, 439.1265, 135.0433, 99.0063, 310.0387, 310.0702, 279.6581	0.67	Regaloside b	C20H26O11	*Lilium lancifolium*
29	NEG	16.96	[M-H]-	595.2041	595.2032	59.0114, 369.1324, 135.0434, 103.8817, 179.0338, 463.3586, 154.4666, 581.0075, 129.2573, 86.3696	1.43	Orobanchoside	C29H36O16	*Pogostemon cablin*
30	NEG	17.76	[M-e]-	500.1698	500.1678	320.093, 292.0963, 86.0974, 70.0664	4.03	3-(3,4-dimethoxyphenyl) -2,8-dimethoxy -6-[(2r,3r, 4r,5r,6r)	C26H30O11	*Pogostemon cablin*

								-3,4,5-tri hydroxy -6-(hydro xymethyl)oxan -2-yl]oxy -4h-napht halen -1-one		
31	NEG	18.4 1	[M-H]$^{-}$	651.19 40	651.193 1	475.1465, 161.0226, 109.0272, 651.1989, 167.0332, 149.0231, 329.088, 242.0068, 160.0147, 228.1125	1.38	Campneo side	C30H38O16	*Pogostemon cablin*
32	NEG	19.4 7	[M-H]$^{-}$	579.20 88	579.208 3	466.4382, 285.2582, 469.4026, 135.8494, 61.6657, 70.4419	0.89	Acteoside /verbasco side/kusa ginin	C29H36O15	*Pogostemon cablin*
33	NEG	19.7 8	[M-H]$^{-}$	267.16 02	267.160 2	267.1596, 57.0324, 267.1326, 226.9941, 178.9916, 79.9551, 247.839, 125.0952, 121.3698, 201.0706	0.24292 5422	10alpha-h ydropero xy -guaia-1, 11-diene	C15H24O2	*Pogostemon cablin*
34	POS	20.2 1	[M+H]$^{+}$	382.12 76	382.128 5	352.0815, 177.0543, 145.0281, 382.1277, 367.1036, 81.071, 338.1016, 324.0863, 117.0333, 95.0865	-2.44	3-desmet hylcolchi cine	C21H23NO6	*Lilium lancifolium*
35	POS	20.6 2	[M+H]$^{+}$	366.13 28	366.133 6	351.1082, 366.1334, 350.103, 336.0855, 335.0788, 308.0911, 307.0829, 149.3246, 250.4099, 91.0548	-2.25	Berlambi ne	C20H17NO5	*Coptis chinensis*
36	NEG	21.2	[M+FA-	505.13	505.135	329.1037, 165.0181, 71.0116, 65.0009,	1.05	Irisolidon	C23H24O10	*Pogostemon*

		8	H]$^-$	57	2	59.0114, 97.0269, 447.7074, 210.5989, 344.7131, 178.4899		e-7-o-α-l-rhamnopyranoside		*cablin*
37	NEG	21.83	[M-H]$^-$	477.1040	477.1038	299.0198, 165.0179, 271.0246, 134.0356, 314.0432, 315.0884, 315.0503, 477.1042, 149.0592, 244.689	0.26	Wogonoside	C22H20O11	*Lilium lancifolium*
38	NEG	23.17	[M-H]$^-$	331.0825	331.0823	178.9978, 166.0263, 193.0125, 151.0016, 138.0303, 175.2528, 165.0181, 74.7958, 191.6293, 91.1336	0.39	Ombuin	C17H14O7	*Pogostemon cablin*
39	POS	23.20	[M+H]$^+$	521.1643	521.1654	359.1116, 301.069, 344.089, 329.0666, 70.0663, 91.5838, 90.7518, 213.1456, 330.6038, 162.6612	-1.95	6-o-e-feruloylajugol	C25H32O12	*Coptis chinensis*
40	NEG	23.40	[M-H]$^-$	301.0716	301.0718	135.0432, 165.0182, 301.0735, 65.0007	-0.43	Genkwanin	C16H12O5	*Pogostemon cablin*
41	NEG	23.44	[M-H]$^-$	461.1092	461.1089	283.0254, 255.0303, 298.0499, 461.1101, 299.0566, 227.0338, 213.7502, 349.9243, 101.4048	0.50	Scutellarein-5-galactoside	C21H20O11	*Lilium lancifolium*
42	NEG	24.53	[M-H]$^-$	401.1453	401.1453	139.0745, 99.0065, 401.1458, 139.0847, 261.061	-0.08	Regaloside a	C18H24O10	*Lilium lancifolium*
43	POS	25.23	[M+H]$^+$	347.1116	347.1125	151.0755, 167.0338, 163.0386, 135.0434, 209.0443, 273.1115, 301.1052, 122.447, 270.0898, 287.4276	-2.65	5-hydroxy-7,3‘,4'-trimethoxyflavanone	C18H18O6	*Pogostemon cablin*
44	POS	28.03	[M+H]$^+$	361.1272	361.1282	167.0337, 163.0385, 91.5773, 70.0663, 57.0713	-2.82	Retusin	C19H18O7	*Pogostemon cablin*
45	NEG	28.6	[M-H]$^-$	247.13	247.134	61.9859, 203.1431, 247.1351, 115.0187,	-1.29	Magnogr	C15H22O4	*Coptis*

		9		37	0	247.0011, 184.0011, 176.9933, 116.9261, 140.9945, 139.9117		andiolide		*chinensis*
46	POS	31.39	$[M+H]^+$	345.0961	345.0969	345.0952, 329.065, 240.2316, 57.0712, 330.0715, 71.0867, 287.0537, 301.0712, 85.102, 109.1015	-2.34	Pachypodol	C18H16O7	*Pogostemon cablin*
47	POS	31.75	$[M+H]^+$	299.0906	299.0914	299.0906, 256.0724, 284.0675, 81.0709, 67.0554, 69.071, 95.0862, 55.0557, 57.0712, 93.0706	-2.60	7-hydroxy-5,8-dimethoxyflavone	C17H14O5	*Lilium lancifolium*
48	POS	32.40	$[M+H]^+$	315.0853	315.0863	315.0854, 81.0709, 300.0608, 272.0673, 67.0555, 95.0863, 69.071, 114.0725, 57.0713, 93.0707	-3.13	3,5-dihydroxy-4',7-dimethoxyflavone	C17H14O6	*Pogostemon cablin*
