# Supplementary material for: Mechanism of Lian-Huo-Hua-Zhuo Formula in Alleviating Gastric Mucosal Inflammation in a Mouse Model of Chronic Atrophic Gastritis by Inhibiting the IL-17 Signaling Pathway
Source: Pharmaceuticals (Basel). 2026 Jul 5;19(7):1043. doi: 10.3390/ph19071043 (PMC13415115; doi:10.3390/ph19071043)
Supplement: Supplementary file 1 [file pharmaceuticals-19-01043-s001.zip › Supplementary Table S2.pdf]

Identification of the upstream primer：-S（sense primer）

Identification of the downstream primer：-A（antisense primer）

Primer Information	Primer Name	Primer Sequence (5'-3')	Fragment Length（bp）	Annealing Temperature（℃）
NM_001278601.1	M-TNFα(5)-S	CCGTCAGCCGATTTGCTATCT	232	60
TNF-α	M-TNFα(5)-A	GCAATGACTCCAAAGTAGACCTG		60
NM_008337.4	M-Ifng（3）-S	GAGGTCAACAACCCACAGGT	136	60
IFN-Y	M-Ifng（3）-A	GGGACAATCTCTTCCCCACC		60
NM_008361.4	M-il1β(1)-S	AGGCTCCGAGATGAACAACAAA	206	60
IL-1β	M-il1β(1)-A	GTGCCGTCTTTCATTACACAGGA		60
NM_031168.2	M-Il6-S	CCCCAATTTCCAATGCTCTCC	141	60
IL-6	M-Il6-A	CGCACTAGGTTTGCCGAGTA		60
NM_001365067.1	M-NFκB P65（2）-S	GCAGAAAGAAGACATTGAGGTGTAT	229	60
NF-κB	M-NFκB P65（2）-A	GCGATCATCTGTGTCTGGCA		60
NM_010552.3	M-IL17A(3)-S	TCCACCGCAATGAAGACCCT	104	60
IL-17	M-IL17A(3)-A	CATGTGGTGGTCCAGCTTTCC		60

NM_007393.3	M-β-actin-S	GTGACGTTGACATCCGTAAAGA	287	60
β-actin	M-β-actin-A	GTAACAGTCCGCCTAGAAGCAC		60
